# A murine model of Lyme disease demonstrates that *Borrelia burgdorferi* colonizes the dura mater and induces inflammation in the central nervous system

**DOI:** 10.1371/journal.ppat.1009256

**Published:** 2021-02-01

**Authors:** Timothy Casselli, Ali Divan, Emilie E. Vomhof-DeKrey, Yvonne Tourand, Heidi L. Pecoraro, Catherine A. Brissette

**Affiliations:** 1 Department of Biomedical Sciences, University of North Dakota, School of Medicine and Health Sciences, Grand Forks, North Dakota, United States of America; 2 Department of Surgery, University of North Dakota, School of Medicine and Health Sciences, Grand Forks, North Dakota, United States of America; 3 Veterinary Diagnostic Laboratory, North Dakota State University, Fargo, North Dakota, United States of America; University of Montana, UNITED STATES

## Abstract

Lyme disease, which is caused by infection with *Borrelia burgdorferi* and related species, can lead to inflammatory pathologies affecting the joints, heart, and nervous systems including the central nervous system (CNS). Inbred laboratory mice have been used to define the kinetics of *B*. *burgdorferi* infection and host immune responses in joints and heart, however similar studies are lacking in the CNS of these animals. A tractable animal model for investigating host-*Borrelia* interactions in the CNS is key to understanding the mechanisms of CNS pathogenesis. Therefore, we characterized the kinetics of *B*. *burgdorferi* colonization and associated immune responses in the CNS of mice during early and subacute infection. Using fluorescence-immunohistochemistry, intravital microscopy, bacterial culture, and quantitative PCR, we found *B*. *burgdorferi* routinely colonized the dura mater of C3H mice, with peak spirochete burden at day 7 post-infection. Dura mater colonization was observed for several Lyme disease agents including *B*. *burgdorferi*, *B*. *garinii*, and *B*. *mayonii*. RNA-sequencing and quantitative RT-PCR showed that *B*. *burgdorferi* infection was associated with increased expression of inflammatory cytokines and a robust interferon (IFN) response in the dura mater. Histopathologic changes including leukocytic infiltrates and vascular changes were also observed in the meninges of infected animals. In contrast to the meninges, we did not detect *B*. *burgdorferi*, infiltrating leukocytes, or large-scale changes in cytokine profiles in the cerebral cortex or hippocampus during infection; however, both brain regions demonstrated similar changes in expression of IFN-stimulated genes as observed in peripheral tissues and meninges. Taken together, *B*. *burgdorferi* is capable of colonizing the meninges in laboratory mice, and induces localized inflammation similar to peripheral tissues. A sterile IFN response in the absence of *B*. *burgdorferi* or inflammatory cytokines is unique to the brain parenchyma, and provides insight into the potential mechanisms of CNS pathology associated with this important pathogen.

## Author summary

Lyme disease is a result of inflammation-induced tissue pathology in response to infection with *Borrelia burgdorferi*. A major barrier to progress in understanding the neurologic manifestations of Lyme disease has been a lack of a tractable laboratory animal model to evaluate the mechanisms of central nervous system pathogenesis. Here we addressed this barrier by characterizing for the first time the kinetics of *Borrelia burgdorferi* colonization in the meninges of laboratory mice, and the resulting immune responses in both the meninges and brain. We demonstrate the presence and kinetics of live spirochetes in the meninges of infected mice, and show that meningeal infection is a general phenomenon shared by several Lyme disease *Borrelia* species. Meningeal colonization was associated with infiltrating leukocytes and local changes in immune response genes previously shown to be associated with Lyme disease pathology. Intriguingly, we show increased expression of immune response genes including those involved in antigen presentation in the brain, despite a lack of detectable infiltrating bacteria or leukocytes in this tissue. Overall, these findings characterize the central nervous system responses to *Borrelia burgdorferi* infection in a cost-effective and genetically robust animal model, and provide insights into the mechanisms of neuropathologies associated with Lyme disease.

## Introduction

Lyme disease (LD), which is caused by infection with the bacterial pathogen *Borrelia burgdorferi* and related species, is a prevalent and continually emerging vector-borne disease throughout North America and Europe [1,2]. Disseminated infection can lead to a number of subacute and persistent inflammatory pathologies affecting the joints (arthritis), heart (carditis and heart block), and nervous systems including the central nervous system (CNS) [3–6]. CNS manifestations can include lymphocytic meningitis, radiculoneuritis, and cranial neuritis. More serious complications during late persistent infection can include encephalitis, and vasculitis [4,6].

Inbred laboratory mice have served as effective models to characterize *B*. *burgdorferi* infection kinetics, as well as the host immune responses involved in pathogen burden control and inflammatory pathology. After initial infection by either needle inoculation or tick transmission, spirochetes disseminate throughout the animal and colonize peripheral tissues including skin, joints, and heart [7,8]. Peripheral tissue colonization is persistent in the absence of antibiotic treatment. Infection of disease-susceptible C3H mice results in subacute arthritis and carditis characterized by joint swelling and histopathological manifestations similar to human disease including leukocyte infiltration [9,10].

Previous studies using a variety of laboratory mouse backgrounds and co-culture models have provided a detailed picture of the differential roles of the host immune responses to infection. *B*. *burgdorferi* and its components can induce host cell production of innate and T cell-mediated inflammatory cytokines as well as chemokines for monocytes, polymorphonuclear leukocytes (PMNs), and lymphocytes both in vitro and during murine infection [11–14]. The innate inflammatory cytokine response is mediated largely through Toll-like receptor (TLR)-2/MyD88/TRIF signaling and NF-κB [15–20], which is essential for efficient control of spirochete burden in conjunction with a lymphocyte-mediated adaptive anti-*B*. *burgdorferi* response [18,19,21,22]. In contrast, inflammatory pathology does not require TLR-2 signaling, B cells, or T cells, but is perpetuated by a robust early interferon (IFN) response in disease-susceptible mice [14,18,19,21–26]. Interestingly, both Type I and Type II IFN contribute to the early induction of IFN-stimulated genes (ISGs); however, Type II IFN and STAT1 are dispensable for murine arthritis development, whereas blockage of Type I IFN signaling leads to reduced joint pathology [23,27–29]. The role of IFN signaling in murine Lyme carditis is less well understood; however, the signals required for disease manifestation appear to be distinct in the heart and joint [28,30,31].

Despite the utility of the murine model for investigating Lyme arthritis and carditis, similar studies on the kinetics of *B*. *burgdorferi* colonization and the resulting host responses are lacking in the CNS of these animals. Given the neurological sequelae associated with *B*. *burgdorferi* infection in addition to arthritis and carditis in Lyme disease patients, a tractable animal model for understanding host-pathogen interactions in the CNS is needed [32]. Previously, we reported that *B*. *burgdorferi* colonizes the dura mater of C3H mice during late disseminated infection, with an associated increase in dura T cell numbers [33]. In the current study, we characterized *B*. *burgdorferi* colonization kinetics in the dura mater during early and subacute infection (7 and 28 days post-infection, respectively), as well as the associated host immune responses in the dura mater and brain parenchyma, as these timepoints have been demonstrated to most consistently replicate inflammatory pathology in murine models of Lyme disease [9,10].

Overall, we report that *B*. *burgdorferi* routinely colonizes the meninges in laboratory mice during early and subacute infection, and induces similar localized inflammatory gene expression profiles as other peripheral tissues as well as histopathological changes. A sterile IFN response in the absence of *B*. *burgdorferi* or inflammatory cytokines is unique to the brain parenchyma, and provides insight into the potential mechanisms of inflammatory CNS pathology associated with this important pathogen.

## Results

### *B*. *burgdorferi* colonize the dura mater during early and subacute infection

**Fig 1:**
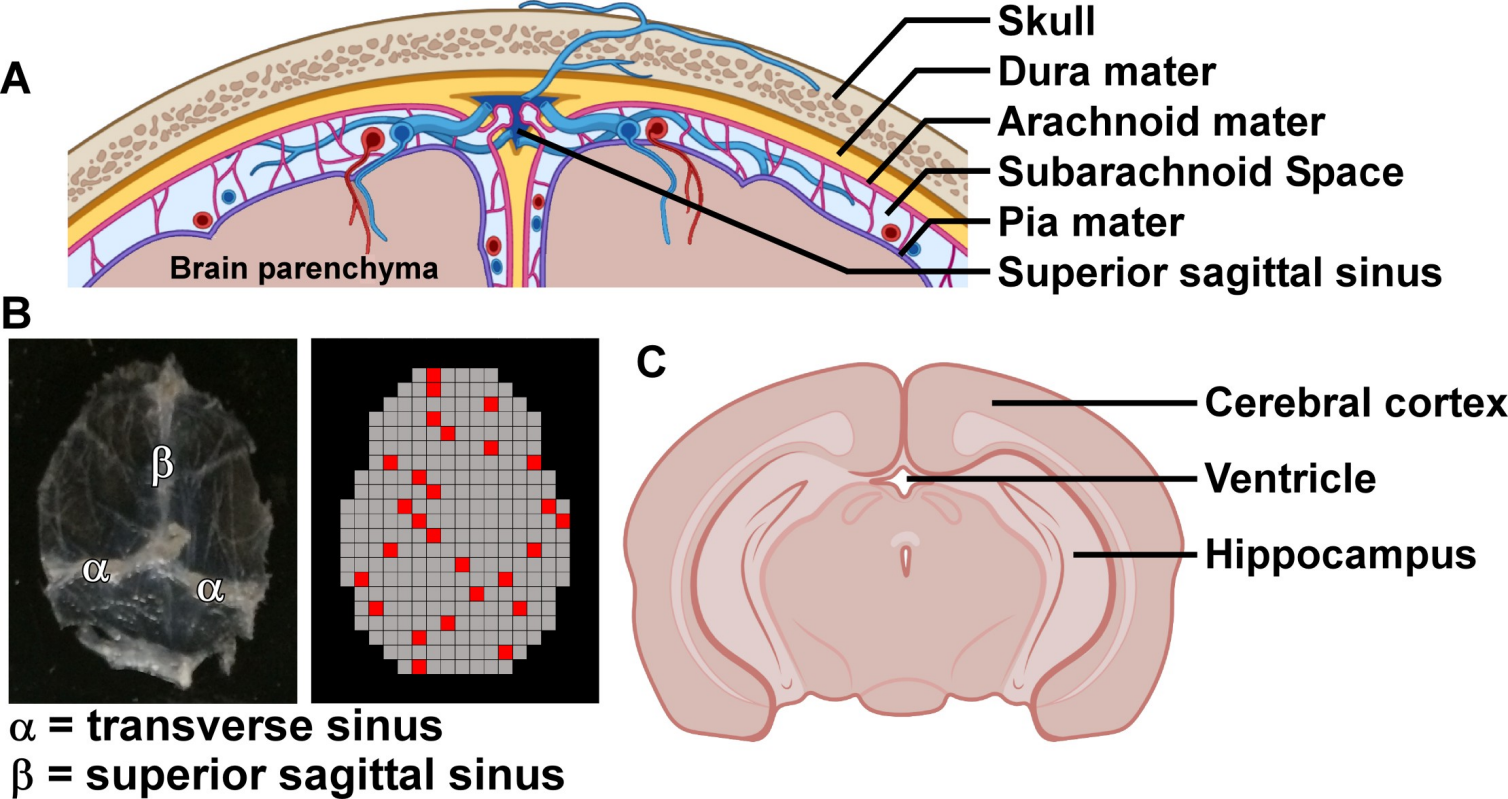
Structure of CNS anatomy relevant to this study. **A.** Schematic coronal view showing the structure of the meninges associated with the calvaria. Three layers of the meninges including the dura mater (yellow), arachnoid mater (pink), and pia mater (purple) separate the brain parenchyma from the skullcap as shown. Beneath the arachnoid mater lies the subarachnoid space, through which cerebrospinal fluid (CSF) flows. The meninges are highly perfused by blood vessels (arteries shown in red, veins shown in blue), and blood to the dura mater is drained by venous sinuses, including the superior sagittal sinus as indicated. **B.** Left panel: Example of dura mater isolated after craniotomy and removal from the calvaria, as used for spirochete counting, RNA extraction, and flow cytometry. Dural venous sinuses are labelled. Right panel: Schematic representation of the grid pattern used for enumerating spirochetes by manual counting using gridded coverslips (0.36mm^2^ grid squares). Samples with more than 1000 positive events were counted using systematic random sampling, by randomly choosing a starting square, followed by counting every tenth square. An example counting scheme is shown using red grid squares. All counts were normalized to a total area of 96.48mm^2^ (268 squares). **C.** Schematic coronal view showing the structures of brain parenchyma used for RNA extraction (cerebral cortex, hippocampus). The location of the third ventricle is also indicated. Choroid plexus is located within the ventricles (not shown). Figs **A** and **C** were created using modified images from BioRender.com.

Based on our previous finding that *B*. *burgdorferi* isolate 297 (Bb_297) colonized the dura mater of C3H mice during late disseminated infection (75 days post-infection) [33], we set out to determine the kinetics of dura mater colonization during early and subacute infection. Mice were initially infected with 10^6^ bacteria by subdermal needle inoculation in the dorsal thoracic skin. At 3–28 days post-infection, mice were perfused transcardially with 4mL PBS to remove circulating blood and spirochetes, and calvaria with attached dura mater were removed. Following craniotomy, dura samples were isolated from the calvaria and assayed by fluorescence-immunohistochemistry (f-IHC) to identify the presence, location, and quantity of spirochetes (see Fig 1 for schematic representation of meningeal anatomy, and example of isolated calvaria-associated dura mater).

**Fig 2:**
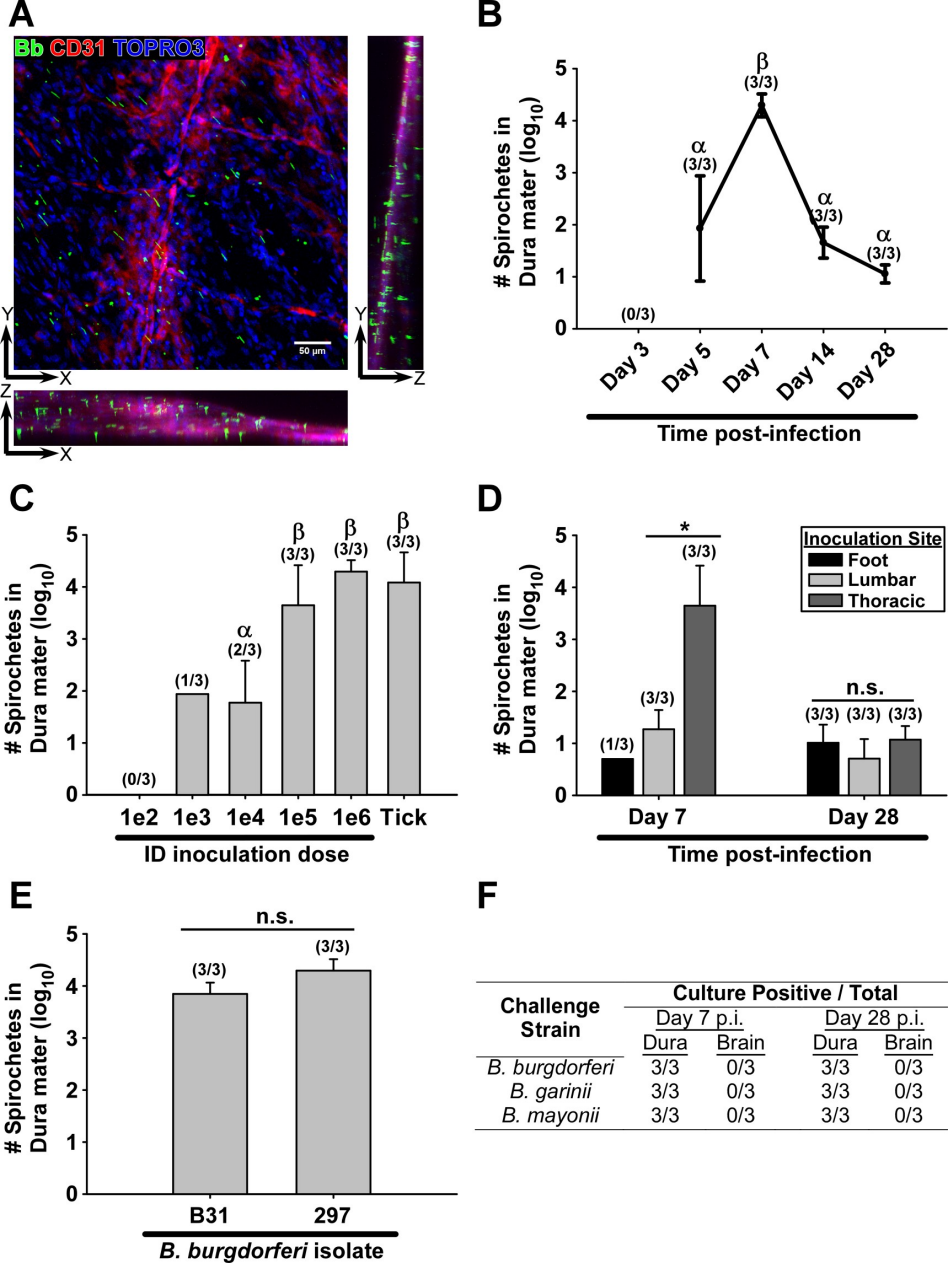
*burgdorferi* infects the dura ubiquitously during early infection. ***B*. A.** Representative confocal z-series showing *B*. *burgdorferi* (Bb, green), blood vessels (CD31, red), and nucleated cells (TOPRO3, blue) in the dura mater of C3H mice on day 7 of infection with 10^6^ spirochetes. X-axis rotation (XZ) and Y-axis rotation (ZY) of image are also shown. **B.** Number of intact spirochetes in the dura mater (log_10_ mean ± s.d.) at various time points after infection with 10^6^ spirochetes. Symbols α and β indicate statistically different groups (p ≤ 0.003; α = 0.05) as determined by one-way ANOVA followed by all pairwise multiple comparison (Holm-Sidak). **C.** Number of spirochetes in dura mater (log_10_ mean ± s.d.) on day 7 of infection using increasing needle inoculation doses as well as tick transmission (10 ticks per mouse). Symbols α and β indicate statistically different groups (0.021 < p < 0.048; α = 0.05) as determined by one-way ANOVA followed by all pairwise multiple comparison (Holm-Sidak). **D.** *B*. *burgdorferi* burden in the dura (log_10_ mean ± s.d.) based on site of inoculation (foot, lumbar, thoracic), and duration of infection (day 7, day 28) of mice infected subdermally with 10^6^ spirochetes. Asterisk indicates significant difference as determined using Student’s t-test (p = 0.009; α = 0.05). “n.s” denotes no statistical significance by one-way ANOVA (p = 0.4; α = 0.05). **E.** Bacterial burden in the dura on day 7 of infection after inoculation with 10^6^ *B*. *burgdorferi* strain B31, or strain 297. “n.s” denotes no statistical significance by Student’s t-test (p = 0.07; α = 0.05). In **(B-E)**, Spirochetes were counted in dura mater by systematic counting using f-IHC, epifluorescence microscopy, and gridded coverslips, as described in Materials and Methods and in Fig 1B. Counts shown are normalized to the approximate area of calvaria-associated dura mater after craniotomy (96.5mm^2^). Number of dura samples with detectable spirochetes / total n are listed for each group above the bar. Conditions with less than two positive dura samples were omitted from statistical analysis. **F.** Number of isolated dura samples resulting in positive cultures in BSK medium from mice infected for 7–28 days with *B*. *burgdorferi* isolate 297, *B*. *garinii*, or *B*. *mayonii* as determined using darkfield microscopy.

**Fig 3:**
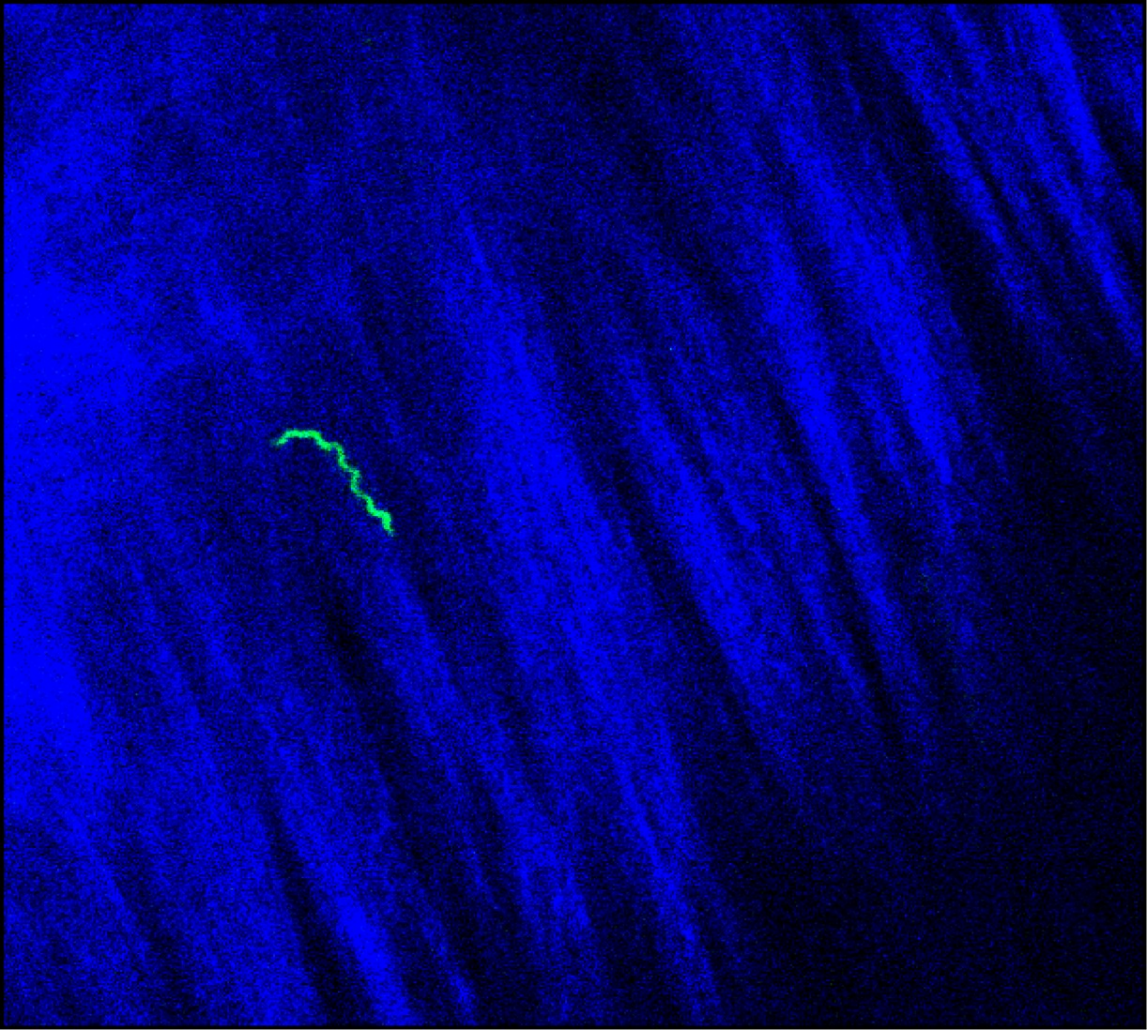
*B*. *burgdorferi* in the dura mater are extravascular and motile. Multiphoton image of ex vivo dura mater from C3H mouse after infection with GFP-Bb_297 for 7 days. *B*. *burgdorferi* is shown in green; collagen (second harmonics) shown in blue. Imaging parameters: Wavelength = 910 nm, pixel resolution = 135. See S1 Movie for image series movie showing spirochete motility.

Whole-mount dura mater stained with anti-*B*. *burgdorferi* antibodies from uninfected mice or 3-day infected mice did not show the presence of intact spirochetes, whereas spirochetes were identified at all later infection timepoints (Fig 2). Spirochetes were ubiquitous throughout the dura mater (Fig 2A), and were largely not associated with nearby blood vessels. Bacterial burden peaked at day 7 post-infection, and declined by days 14 and 28 to levels similar to that seen at day 75 post-infection [33] (Fig 2B); see Fig 1B for example of bacterial enumeration strategy. Multiphoton imaging of the dura mater of mice infected with Green Fluorescent Protein-expressing Bb_297 (Bb_297-GFP) confirmed spirochetes are extravascular, alive, and motile (Fig 3 and S1 Movie). Despite the abundance of Bb_297 in the dura mater, no spirochetes were observed in the brain parenchyma (not shown), indicating that spirochetes were limited to the meninges in the CNS of mice.

Inoculation dose is an important consideration during experimental infection of laboratory animals. As our initial experiments were done using 10^6^ bacteria, we repeated the experiment with decreasing inoculation doses of Bb_297. At day 7 post-infection, no spirochetes were observed in the dura mater of mice inoculated with 10^2^ bacteria, whereas inoculation doses of 10^3^ and 10^4^ spirochetes resulted in only 1/3 and 2/3 positive dura, respectively, with relatively low levels of detectable spirochetes (Fig 2C). All mice had detectable spirochetes in the dura mater at day 7 post-infection after inoculation with 10^5^−10^6^ bacteria, with higher bacterial burdens compared to the lower inoculation doses (Fig 2C).

Given the dose-dependent nature of dura colonization by needle inoculation, we repeated the experiment using mice infected by nymphal tick transmission to determine the relevance after exposure from the natural vector (10 infected ticks per mouse placed on dorsal thoracic/head region). Seven days post-transmission feeding, dura spirochete burdens in all mice were similar to that seen using a needle inoculation dose of 10^6^ bacteria (Fig 2C). Therefore, all future experiments were carried out using subdermal needle inoculation with 10^6^ spirochetes to mimic dura colonization observed after tick transmission.

We also examined the effects of inoculation site on dura colonization. Mice were infected by needle inoculation in either the footpad, dorsal lumbar skin, or dorsal thoracic skin. At day 7 post-infection there was a marked difference in dura colonization efficiency between inoculation sites, with higher spirochete burdens observed after infection at sites more proximal to the dura mater (Fig 2D). This difference was abrogated by day 28 post-infection, indicating that only the initial peak in burden is dependent on inoculation site, and not the ability to persistently colonize this tissue.

Bb_297 is a clinical isolate from the CSF of a patient diagnosed with neuroborreliosis [34]. To determine if additional Lyme disease *Borrelia* isolates are capable of dura colonization in mice, we repeated the experiment with the *B*. *burgdorferi* tick isolate B31 [35]. No difference was seen in bacterial burden in the dura mater between *B*. *burgdorferi* sensu stricto (s.s.) isolates (Fig 2E). We were also able to culture both *B*. *garinii* (a European *B*. *burgdorferi* sensu lato (s.l.) species) as well as *B*. *mayonii* (a North American *B*. *burgdorferi* s.l. species) from the dura mater of perfused mice at both day 7 and day 28 post-infection; however, brain cultures from all animals were negative [36,37]. Taken together, colonization of the dura mater in mice is a general phenomenon for clinical and tick isolates of *B*. *burgdorferi* s.s., as well as both North American and European isolates of *B*. *burgdorferi* s.l.

### *B*. *burgdorferi* infection leads to leukocyte infiltration in the dura mater

A hallmark of laboratory murine Lyme arthritis and carditis is a subacute leukocytic infiltration between 2–4 weeks post-infection [9,10]. We examined the dura mater and brain parenchyma at 7 days post-infection (during peak spirochete load in the meninges) as well as 28 days post-infection (timepoint of peak Lyme arthritis) for signs of inflammatory cell infiltrate.

**Fig 4:**
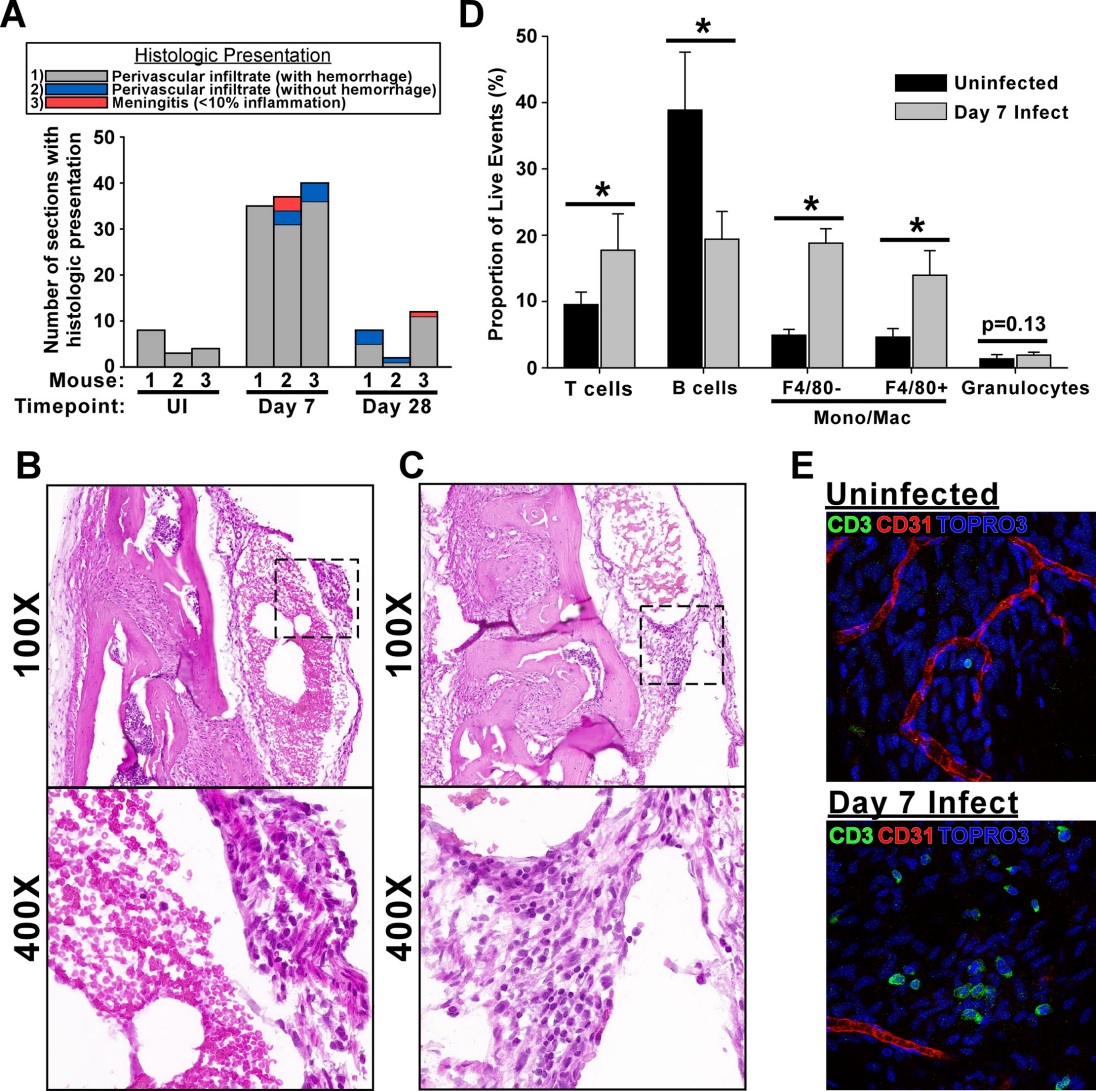
Infection with *B*. *burgdorferi* leads to leukocyte infiltration in the dura mater. **A.** Coronal sections of skullcaps with attached dura were stained with hematoxylin and eosin, and 60 representative sections were systematically evaluated for inflammation. Stacked bargraph shows number of sections from each mouse with histologic presentations as shown in the legend. Timepoint of infection is shown for each mouse. Representative z-series images of perivascular leukocyte infiltrate associated with hemorrhage (**B**) and perivascular infiltrate extending to minimal meningitis (**C**) are shown at 100X and 400X magnification as indicated. Boxed areas from 100X images show areas used for high magnification. See S9–S12 Figs for full resolution images. **D.** Bar graph showing frequencies of immune cell subtypes ± s.d. in the dura mater from day 7 post-infection compared to uninfected controls as determined using flow cytometry (n = 6 per group). Bars represent percentage of live events after gating on cells of interest based on forward and side scatter area (average number of live events ± s.d. = 5524 ± 2643 across all dura samples). Detailed gating strategy for cell subtypes is outlined in S1 Fig. Cell populations were defined as: T cells: CD45+CD3+; B cells: CD45+CD19+; Monocyte/Macrophage-enriched: CD45+CD3-CD19-CD11b+Ly6G-; Granulocyte-enriched: CD45+CD3-CD19-CD11b+Ly6G+. Asterisks indicate statistically different groups (0.003 ≤ p ≤ 0.010; α = 0.05) as determined using Student’s t-test. **E.** Representative confocal images are shown from uninfected and 7-day infected mice. T cells (CD3, green), blood vessels (CD31, red), and nucleated cells (TOPRO3, blue) are shown.

Calvaria with attached dura mater were isolated by craniotomy for histopathologic assessment. Coronal sections stained with hematoxylin/eosin revealed that more than half of all dura sections from 7-day infected mice showed signs of perivascular infiltrate associated with vascular hemorrhage (34.0±2.6 sections; 57%), compared to 5.0±2.6 sections (8%) and 5.6±5.0 (9%) of uninfected and 28-day infected dura, respectively, indicating changes to the meningeal vasculature at day 7 post-infection (Fig 4A–4C). In addition to the observed increased prevalence of vascular hemorrhage in the dura of infected mice, we also noted evidence of perivascular leukocyte infiltrate and/or mild/minimal meningitis in the absence of vascular hemorrhage in sections from 5/6 infected mice, but not in any of the control mice (Fig 4A–4C).

We previously showed dura colonization by *B*. *burgdorferi* during late disseminated infection is associated with an increase in T lymphocytes, however the presence of other leukocytes was not examined [33]. We therefore examined changes in leukocyte populations using flow cytometry on single cell suspensions of the dura mater from 7 day infected and control mice following transcardial perfusion with PBS. Dura mater from infected mice showed a significant increase in CD45+ hematopoietic cells as a proportion of live singlets compared to uninfected controls (34.54% ± 3.49% vs 23.18% ± 5.02%; p = 0.002). Further subtyping (see S1 Fig for detailed gating strategy) showed significant increases in the frequency of populations enriched for T cells (CD45+CD3+CD19-) and monocytes/macrophages (CD45+CD3-CD19-CD11b+Ly6G-), and a significant decrease in the frequency of B cell-enriched population (CD45+CD3-CD19+) in the dura mater from infected mice compared to uninfected controls (Fig 4D). No change was detected in the granulocyte-enriched population (CD45+CD3-CD19-CD11b+Ly6G+) (Fig 4D). The increase in CD3+ cells in infected dura was verified by f-IHC. Examination of uninfected dura showed the presence of scattered CD3+ cells that appeared small and spherical (Fig 4E). Dura from infected animals displayed a marked increase in the number of CD3+ cells, which appeared larger and had morphology/staining profiles consistent with more active/motile cells (Fig 4E).

**Fig 5:**
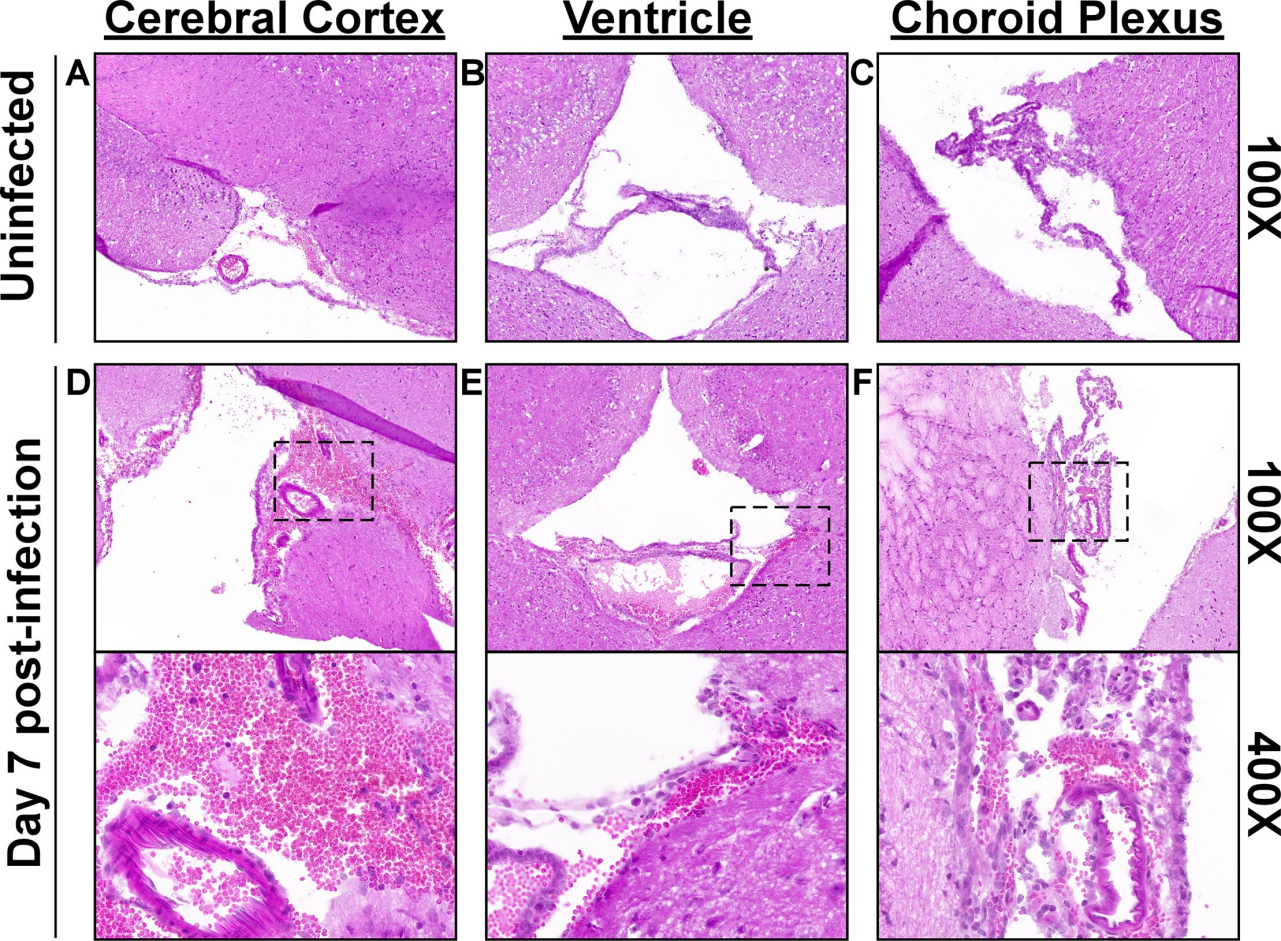
Infection with *B*. *burgdorferi* is associated with perivascular hemorrhage admixed with leukocytes in the leptomeninges and ventricles, without leukocytic infiltration into the brain parenchyma. Uninfected mice showed no microscopic evidence of inflammation in brain sections examined **(A-C)**, while perivascular hemorrhage with attendant leukocytes was observed in the cerebral meninges **(D)**, ventricles extending into the subependymal zone **(E)**, and throughout the choroid plexus **(F)** at day 7 post-infection. Leukocytic infiltration was not noted in the cerebral cortex, hippocampus, or thalamus in either control or infected mice. Infection status is shown to the left of panels, while magnification is shown on the right. Boxed areas from 100X images show areas used for high magnification. See S13–S21 Figs for full resolution images.

Given the observed histopathologic changes in the calvaria-associated dura mater during *B*. *burgdorferi* infection, we next set out to determine evidence of inflammation in the brains isolated from the same animals. Histopathologic analysis of coronal brain sections stained with hematoxylin/eosin did not reveal leukocytic infiltration in the cerebral cortex, hippocampus, or thalamus of either the control or infected mice; consistent with the lack of detectable spirochetes in the parenchyma. Meningeal congestion was occasionally observed in all mice, including control mice, but meningeal perivascular hemorrhage admixed with leukocytes was observed only in infected mice. This perivascular hemorrhage was characterized by extravasated red blood cells admixed with rare to few leukocytes expanding the leptomeninges, especially along the ventral cortex, and choroid plexus or extending into the subependyma of the lateral ventricle (Fig 5). Interestingly, leukocyte infiltrates associated with hemorrhage were seen in the leptomeninges and ventricles of nearly all (99.4%) of the 176 brain sections evaluated on day 7 infected mice compared to 74% (129/174) of the day 28 infected mice.

Taken together, dura colonization by *B*. *burgdorferi* is associated with a localized infiltration of leukocytes of myeloid and lymphoid origin. Additionally, increases in perivascular hemorrhage associated with blood vessels of the dura mater, cerebral meninges, and ventricles, suggest vascular alterations in these tissues in response to *B*. *burgdorferi* infection in mice.

### *B*. *burgdorferi* infection is associated with large scale changes in gene expression in the dura mater and brain parenchyma

#### Overview of gene expression analysis

Studies on gene expression changes in joint and heart tissues have provided insights into the localized host responses to *B*. *burgdorferi* colonization [14,38]; however, gene expression changes in the CNS of mice during *B*. *burgdorferi* infection remain unclear. Therefore, we took an unbiased approach to examine potential changes in the CNS at day 7 post-infection using RNA sequencing (RNA-seq). Prior to euthanasia, mice were anesthetized and transcardially perfused with 4 mL PBS to remove gene expression signatures from circulating blood, followed by 4mL RNAlater.

Lyme disease in humans can be associated with a diffuse encephalopathy including memory problems and other cognitive impairments such as confusion, reduced attention/concentration, mental fatigue, reduced verbal fluency, and others with varying severity [39]. Such functions are largely governed by the cerebral cortex (e.g. attention, perception, awareness, long-term memory), and the hippocampus (e.g. short-term memory). We therefore examined gene expression changes in the dura mater of infected mice where live spirochetes were detected, in addition to the cerebral cortex and hippocampus to reflect specific tissues associated with clinical Lyme disease CNS manifestations.

**Fig 6:**
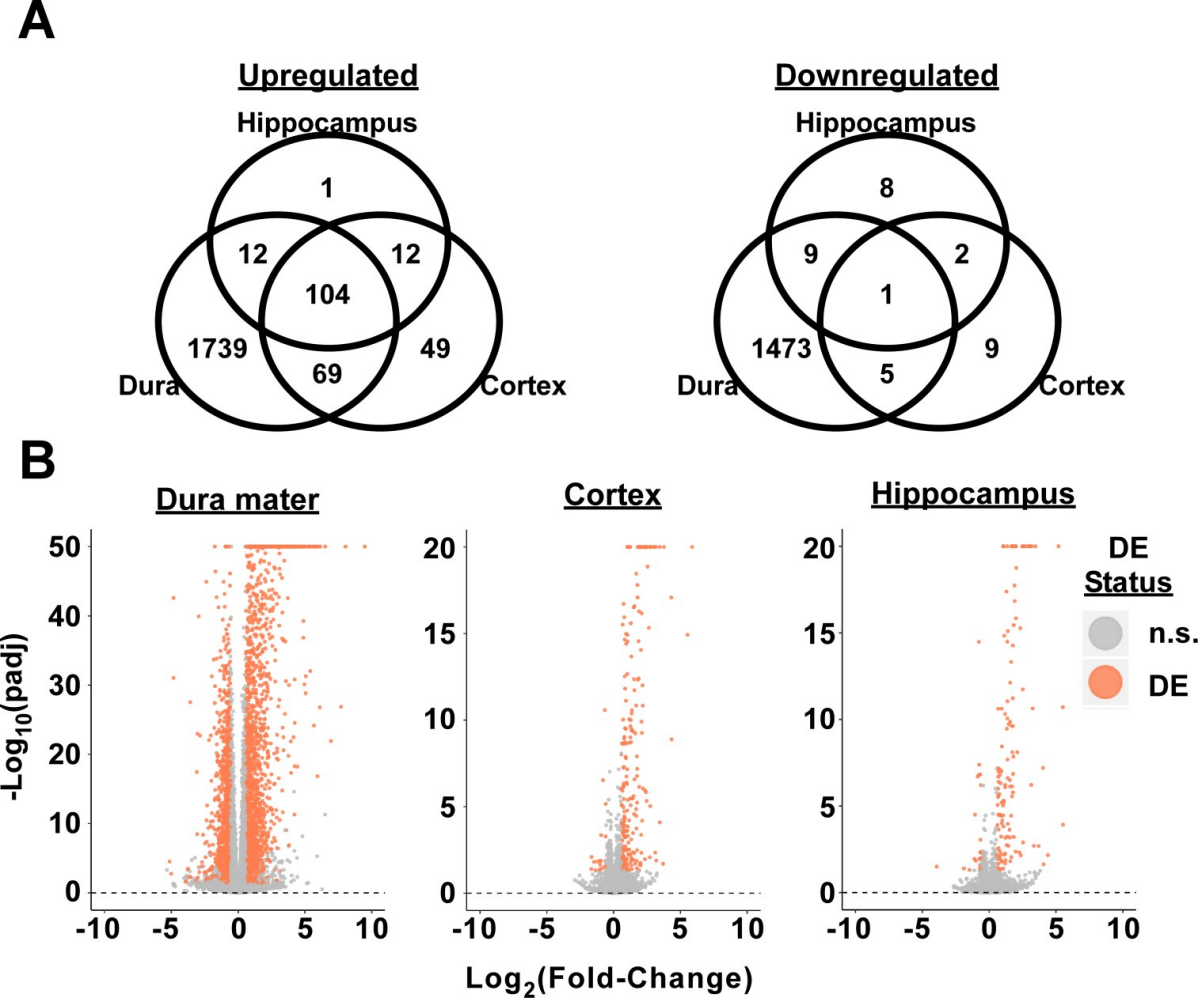
Infection with *B*. *burgdorferi* leads to transcriptome changes in the meninges and brain parenchyma. **A.** Venn diagram showing the number of distinct and common significantly upregulated and downregulated genes in the dura mater, brain cortex, and hippocampus (padj ≤ 0.05, basemean ≥ 20, fold-change ≥ 1.5) of 7-day infected mice compared to uninfected controls (n = 4 per timepoint). **B.** Volcano plots from the three tissues tested comparing -log(padj) to log_2_(fold-change) for all genes. Red color indicates DEGs, while gray indicates genes not significantly different between infected and control mice (n.s.). See S2 Fig for principle component analysis and hierarchical clustering of individual samples.

Over 1,900 and 1,400 genes were significantly upregulated or downregulated in the dura mater of infected mice, respectively (Fold-Change ≥ 1.5, padj ≤ 0.05, basemean > 20) (Fig 6) see S1 Table for complete comparative expression list and individual padj values. Additionally, brain cortex and hippocampus both exhibited a substantial number of differentially expressed genes (DEGs; 258 and 158, respectively) (Fig 6 and S2 and S3 Tables). Although the number of upregulated and downregulated DEGs in the dura mater were comparable (56% vs 44% upregulated vs downregulated, respectively), the majority of DEGs in the brain were upregulated (93% in cortex, 82% in hippocampus). These responses were consistent across biological treatments and replicates (S2 Fig). Collectively, these data show a robust response to *B*. *burgdorferi* colonization in the dura mater as well as changes in gene expression in the brain parenchyma at day 7 post-infection, despite the absence of a local presence of spirochetes or infiltrating leukocytes in the brain.

#### Upregulated genes in the dura mater, cortex, and hippocampus demonstrate profiles consistent with IFN response, whereas upregulation of inflammatory cytokines and chemokines are restricted to the dura mater

The top five most enriched molecular function gene ontology (GO) terms from genes upregulated in the dura mater all pertained to cytokine/chemokine production, activity, or their receptors (S3 Fig panel A). In contrast, GO terms enriched in upregulated genes in both the cortex and hippocampus were related to antigen processing and presentation, double-stranded RNA-binding, and GTPase activity (S3 Fig panels B-C). When only those genes commonly upregulated in all three tissues were considered, a similar GO profile was observed as cortex or hippocampus alone, indicating that these molecular functions were enriched across all tissues tested (S3 Fig panel D).

**Fig 7:**
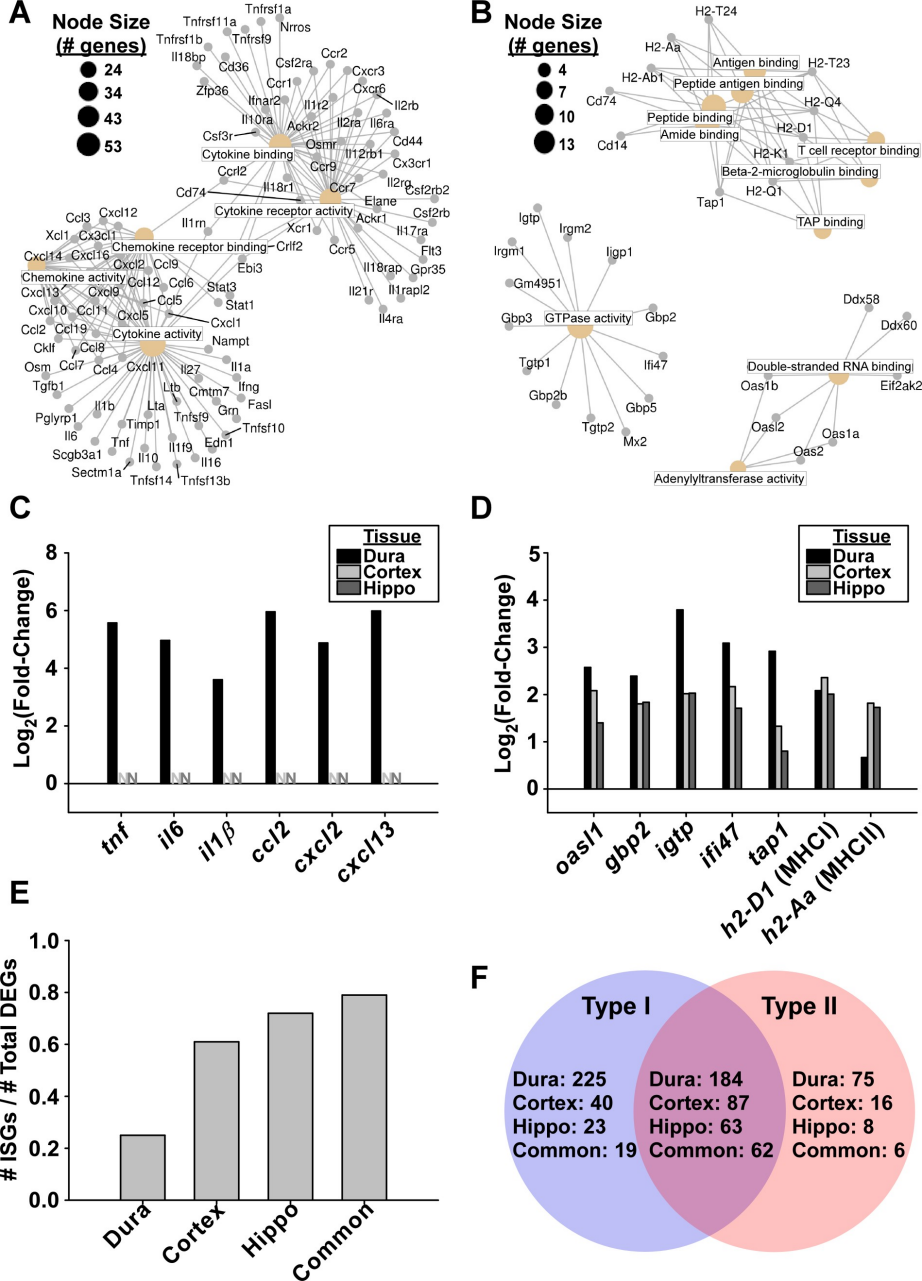
Upregulated genes in the dura mater, cortex, and hippocampus demonstrate profiles consistent with IFN response, whereas upregulation of inflammatory cytokines are restricted to the dura mater. **A.** Cnet plot (generated using ClusterProfiler [101]) showing individual genes associated with the top 5 GO terms upregulated in the RNA-seq dataset from the dura mater of infected C3H mice. Colored nodes represent GO terms, with node descriptions represented as boxed text. Node size correlates with the number of associated genes, as shown in the legend. Individual upregulated genes are represented by gray dots, with lines connecting to the relevant GO terms. **B.** Cnet plot as described in **(A)**, showing top 10 enriched GO terms and associated genes commonly upregulated in all three tissues. **C.** Barplot representation of log_2_(fold-change) from RNA-seq data of selected genes from **(A)**. Bars denote magnitude of change for DEGs, whereas “N” represents genes not differentially expressed, demonstrating the tissue specificity of cytokine response. **D.** Barplot representation from RNA-seq data of selected genes from **(B)**, showing similarity of response in the three tissues. **E.** Proportion of upregulated genes from each tissue as well as those genes commonly upregulated in all tissues that are predicted to be upregulated by IFN [40]. **F.** Venn diagram showing number of upregulated DEGs from each tissue predicted to be stimulated by Type I or Type II IFN [40].

Examination of individual upregulated genes associated with the five most enriched GO terms in the dura mater revealed a number of hallmark inflammatory cytokines induced by *B*. *burgdorferi* including *tnf*, *il6*, and *il1β* (Fig 7A and 7C and S1 Table). Several chemokines of monocytes (*ccl2*, *ccl7*), PMNs (*cxcl1*, *cxcl2*), and lymphocytes (*ccl2*, *ccl7*, *ccl19*, *cxcl9*, *cxcl10*, *cxcl13*) were also upregulated. The majority of these genes were upregulated greater than 10-fold in the dura mater, however were not differentially expressed in the RNA-seq dataset in the cortex or hippocampus (Fig 7C and S1–S3 Tables). As an inflammatory cytokine response is thought to be initiated in response to *B*. *burgdorferi* antigens through TLR signaling and NF-κB activation [15–20], it is perhaps not surprising that upregulation of these genes is largely limited to the dura mater where spirochete burden is prominent. Indeed, we observed significant upregulation of TLR genes *tlr1*, *tlr2*, *tlr6*, *tlr7*, *tlr8*, and *tlr9* as well as adaptor molecules including *myd88* (S1 Table), and Signaling Pathway Impact Analysis (SPIA) revealed activation of both TLR (pGFWER = 1.30E-06) and NF-κB (pGFWER = 1.15E-16) signalling pathways exclusively in the dura mater (S4 and S5 Figs).

A subset of 104 genes were found to be similarly upregulated in all three tissues tested. Examination of these common upregulated genes associated with the 10 most enriched GO terms revealed a number of IFN-inducible GTPases and signaling molecules including *oasl1*/*2*, *gbp2*/*3*/*5*, *igtp*, as well as several genes involved in antigen processing/presentation by both MHCI and MHCII (e.g. *H2-Aa*, *H2-D1*, *H2-K1*, *H2-Q1*, *tap1*) (Fig 7B–7D for gene expression; S6 Fig for SPIA). In fact, the majority of upregulated genes in the cortex (61%), hippocampus (72%), and those common to all three tissues (79%) were associated with IFN response, while ISGs constituted 25% of the total number of upregulated genes in the dura mater [40](Fig 7E). ISGs associated with both Type I and Type II IFN were identified in all tested tissues (Fig 7F). Intriguingly, Type II IFN was only detected at low levels in the dura mater, and Type I IFN was not detectable in any tissue type despite a robust IFN response; although genes coding for both Type I and Type II IFN receptors were significantly upregulated in the dura mater (S1 Table).

Gene expression profiles from the dura mater were consistent with our histopathological and flow cytometric findings of an influx of immune cells including an increase in T cells and myeloid leukocytes, with a concurrent decrease in B cells. Expression of endothelial cell adhesion molecules *vcam1* and *icam1* were significantly upregulated in the dura of infected mice (S1 Table). Additionally, SPIA showed activation of the T cell receptor signalling pathway (pGFWER = 8.48E-05) including increased expression of all three CD3 subunits as well as downstream molecules (S1 Table and S7 Fig), whereas a significant decrease was observed for the B cell marker *cd19* (log_2_(fold-change) = -1.49; padj = 5e-06). Expression of *ptprc* (CD45) (log_2_(fold-change) = 1.83; padj = 1e-32) *itgam* (CD11b) (log_2_(fold-change) = 3.66; padj = 1e-50) and *emr1* (f4/80) (log_2_(fold-change) = 3.15; padj = 5e-75) were highly upregulated in infected dura, as well as genes encoding Ly6C (*ly6c1*, *ly6c2*), while *itgax* (CD11c) and Ly6G genes were not significantly altered, consistent with an increase in monocytes/macrophages (S1 Table).

In addition to increased expression of MHC genes in the brain parenchyma, both *itgax* (CD11c) and Ly6c genes were differentially upregulated in the cortex of infected mice, while *itgam* (CD11b) expression levels were only modestly increased (45% upregulation; padj = 0.02). Hippocampus samples showed an upregulation of Ly6c genes in response to infection; however, no difference was detected in expression levels of CD11b or CD11c genes. No significant differences in B cell or T cell marker genes were detected in the brain parenchyma, consistent with a lack of observed infiltrating leukocytes in the parenchyma as determined by histopathologic analysis.

Collectively these data show the presence of two distinct immune profiles in the CNS of infected mice. In the dura mater, the presence of *B*. *burgdorferi* is associated with upregulation of genes consistent with TLR/NF-κB signalling and associated inflammatory cytokines and chemokines in addition to a robust IFN response. In contrast, the brain parenchyma from mice infected with *B*. *burgdorferi* exhibits mainly an IFN response without an associated cytokine gene expression response or evidence of spirochetes in this tissue.

#### Colonization of the dura mater is associated with altered expression of genes associated with the extracellular matrix, cell-adhesion, and wound repair

**Fig 8:**
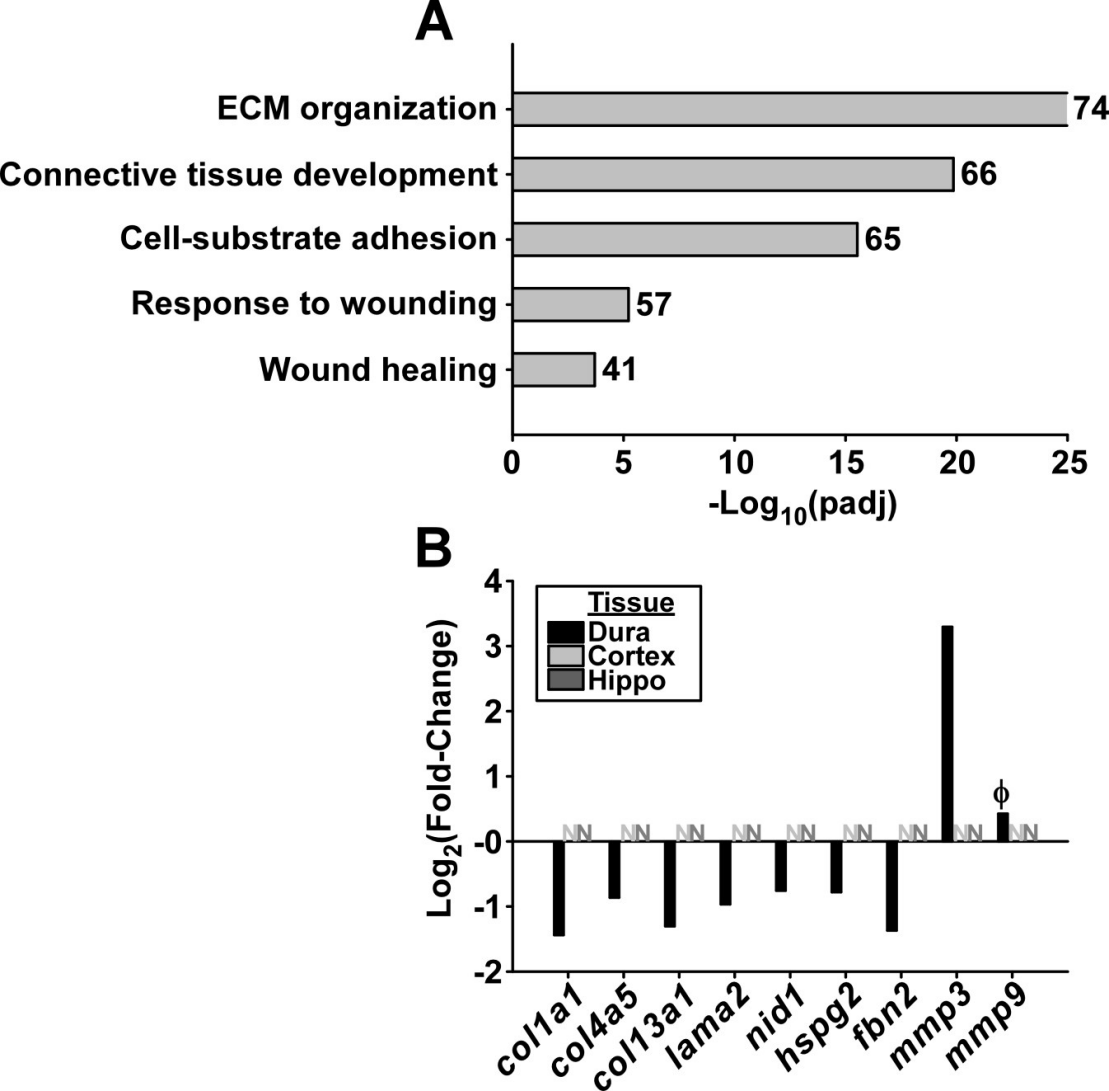
Colonization of the dura mater is associated with downregulation of genes associated with the extracellular matrix. **A.** Selected biological process GO terms enriched in downregulated genes in the dura mater. Numbers to the right of horizontal bars show the number of downregulated DEGs associated with each term. Bar size represents significance of enrichment (-log(padj)). **B.** Log2(fold-change) of selected genes from RNA-seq dataset associated with GO terms from **(A).** Bars represent magnitude of change for differentially expressed genes, while “N” represents genes not significantly differentially expressed in the RNA-seq dataset; demonstrating the tissue specificity of the response. Φ represents statistical significance (p = 0.003), but does not meet the pre-determined fold-change cut-off to be classified as a DEG (fold-change = 1.3; DEG cut-off = 1.5).

Previous studies have shown an inverse correlation between IFN-inducible gene expression and genes associated with wound healing and tissue repair in both murine Lyme arthritis and synovial tissue from Lyme arthritis patients [14,41]. Likewise, biological process GO term enrichment from downregulated genes in the dura mater of infected mice showed genes involved in ECM organization, connective tissue development, cell-substrate adhesion, and wound repair (Fig 8A). Examination of individual genes showed significant downregulation of tissue growth factors (*bmp5*, *bmp7*) and cell-signalling genes (*wnt5b*, *wnt9A*, *adcy5*) involved in tissue repair (S1 Table). Several ECM components including fibrillary collagens (*col1a1*, *col1a2*, *col2a1*, *col3a1*, *col5a1*, *col5a2*, *col5a3*, *col6a2*, *col6a3*, *col9a1*, *col9a2*, *col11a1*, *col11a2*, *col15a1 col16a1*), basement membrane (BM)-associated collagens (*col4a5*, *col4a6*, *col8a1*, *col8a2*), and transmembrane collagens (*col13a1*, *col24a1*, *col25a1*) were also downregulated. Other BM components downregulated in infected dura included perivascular BM-associated laminins (*lama2*, *lama3*, *lamb1*, *lamb3*), nidogen (*nid1*, *nid2*), heparin sulfate proteoglycans (perlecan; *hspg2*) and other ECM components (fibrillin; *fbn1*, *fbn2*) (Fig 8B and S1 Table). Genes coding for the ECM proteins fibronectin (*fn1*) and decorin (*dcn*) were highly expressed in the dura mater; however, expression levels were not altered in response to infection (S1 Table).

The above ECM components affected by *B*. *burgdorferi* infection are the targets of host proteases that allow for tissue remodelling under physiologic or pathologic conditions [42]. The matrix metalloproteinase gene *mmp3* was significantly upregulated in the dura mater of infected mice (Fig 8B), which is involved in tissue reorganization, wound repair, and activation of other MMPs (e.g. MMP9) [42], and has previously shown to be induced by *B*. *burgdorferi* in C3H mice [14,43]. The murine Lyme arthritis-associated *mmp9* was also significantly upregulated in the dura mater of infected mice at day 7 post-infection [14,44,45]; however, the magnitude of expression change did not reach our fold-change cut-off for differential expression calling (padj = 0.003; Fold-change = 1.3; DEG cut-off = 1.5).

Additionally, we found significantly decreased expression of genes encoding gap and adherens junction proteins including several connexins and ZO-1 (*tjp1*) (S1 Table). The endothelial adhesion molecule VE-cadherin gene *cdh5* that is normally expressed in blood and lymphatic vessels of the dura mater was significantly decreased by 44% (p = 3.7e-07), although this did not reach our pre-determined cut-off for differential expression status (S1 Table) [46].

Taken together, these data illustrate large-scale altered expression of genes coding for growth factors, signalling molecules, proteases, and structural proteins involved in ECM remodelling and tissue repair, as well as genes involved in endothelial junction maintenance.

#### Increased expression of inflammatory cytokines persists during disseminated infection, whereas IFN response is limited to early infection

Host gene expression profiles from the RNA-seq dataset at day 7 post-infection in the dura mater and brain parenchyma were confirmed by qRT-PCR of representative genes. Additionally, the presence of live *B*. *burgdorferi* was evaluated by qRT-PCR of the bacterial *flaB* gene. Additional timepoints from 7–56 days post-infection were assessed to determine the kinetics of host responses to infection. The *tnf* gene was chosen as a proxy for inflammatory cytokines, as well as *gbp2* as proxy for ISGs. C*xcl10* expression was also assayed, as this gene has been demonstrated as an ISG, with evidence of non-IFN induction by NF-κB [47].

**Fig 9:**
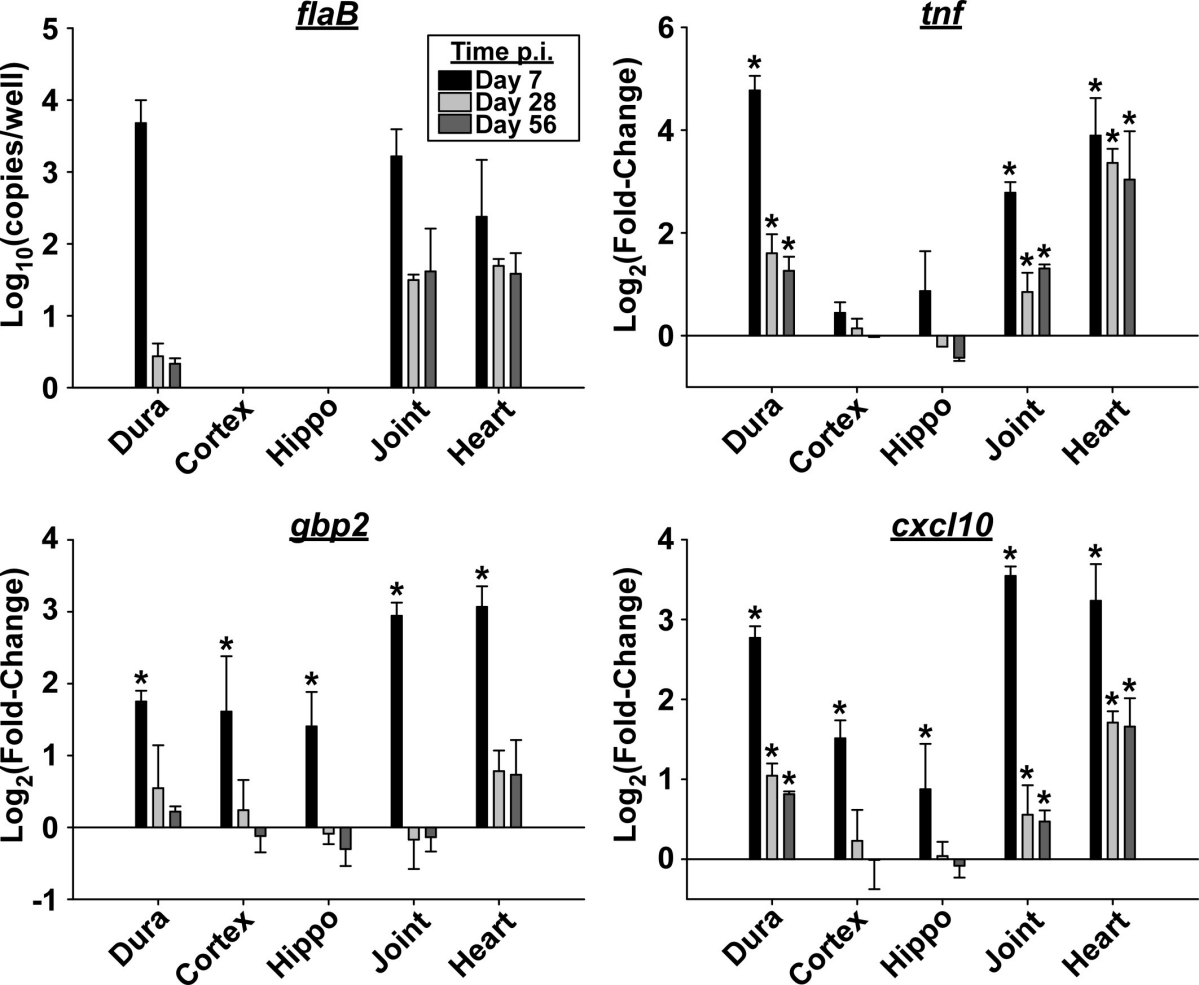
Increased expression of inflammatory cytokines persists during late disseminated infection, whereas IFN response is limited to early infection. qRT-PCR of selected genes from dura mater (dura), cortex, hippocampus (hippo), tibiotarsal joint (joint), and heart tissues from day 7–56 post-infection as shown in the legend (n = 4 per timepoint). The *B*. *burgdorferi* gene *flaB* is displayed as gene copies (log_10_ mean ± s.d.) per reaction containing 12.5ng input cDNA as determined using standard curves. Levels of *flaB* copies were below the limit of detection in the cortex and hippocampus. Host genes *tnf*, *gbp2*, and *cxcl10* are displayed as log_2_(fold-change) (mean ± s.d.) compared to uninfected controls as determined using the ΔΔCt method. Asterisks indicate statistical significance (0.001 ≤ p ≤ 0.026; α = 0.05) using one-way ANOVA followed by Dunnett’s test.

*FlaB* transcripts were readily detected at all timepoints in the dura mater, tibiotarsal joints, and heart tissues, whereas no *flaB* transcript was detectable in either the cortex or hippocampus at any timepoint (Fig 9), consistent with our initial microscopy and culture data. Likewise, *tnf* levels were elevated in the dura mater, joint, and heart tissues at all timepoints compared to uninfected controls, but were not increased in the cortex or hippocampus, supporting our RNA-seq data showing increased cytokine expression correlates with the presence of spirochetes. In contrast, the ISG *gbp2* was elevated in all tissues tested including brain tissues at day 7 post-infection, again in agreement with the RNA-seq dataset. Expression of *gbp2* returned to uninfected levels by day 28 post-infection in all tissues, which is a pattern that has been previously reported for ISGs in joints during murine Lyme arthritis [14]. Cxcl10 was found to be elevated in all tissues at day 7 post-infection, consistent with its induction by IFN. This gene remained elevated in dura, joint, and heart tissue up to 56 days post-infection, however returned to uninfected levels after day 7 post-infection in the brain. This profile of *cxcl10* expression suggests that in addition to early induction in all tissues as part of an IFN response, the persistence of spirochetes in peripheral tissues and the dura mater is sufficient for sustained elevated expression of this gene.

These data show that *B*. *burgdorferi* persistently colonize the dura mater, and induces a sustained inflammatory cytokine response in addition to an early transient IFN response. In contrast, *B*. *burgdorferi* do not colonize the brain parenchyma; however, a transient IFN response is still induced in the cortex and hippocampus of infected animals that returned to uninfected levels as infection persisted.

### T cells and B cells are necessary for efficient reduction of *B*. *burgdorferi* load during persistent infection in the dura mater, however are not required for leukocyte infiltration or upregulation of ISGs

**Fig 10:**
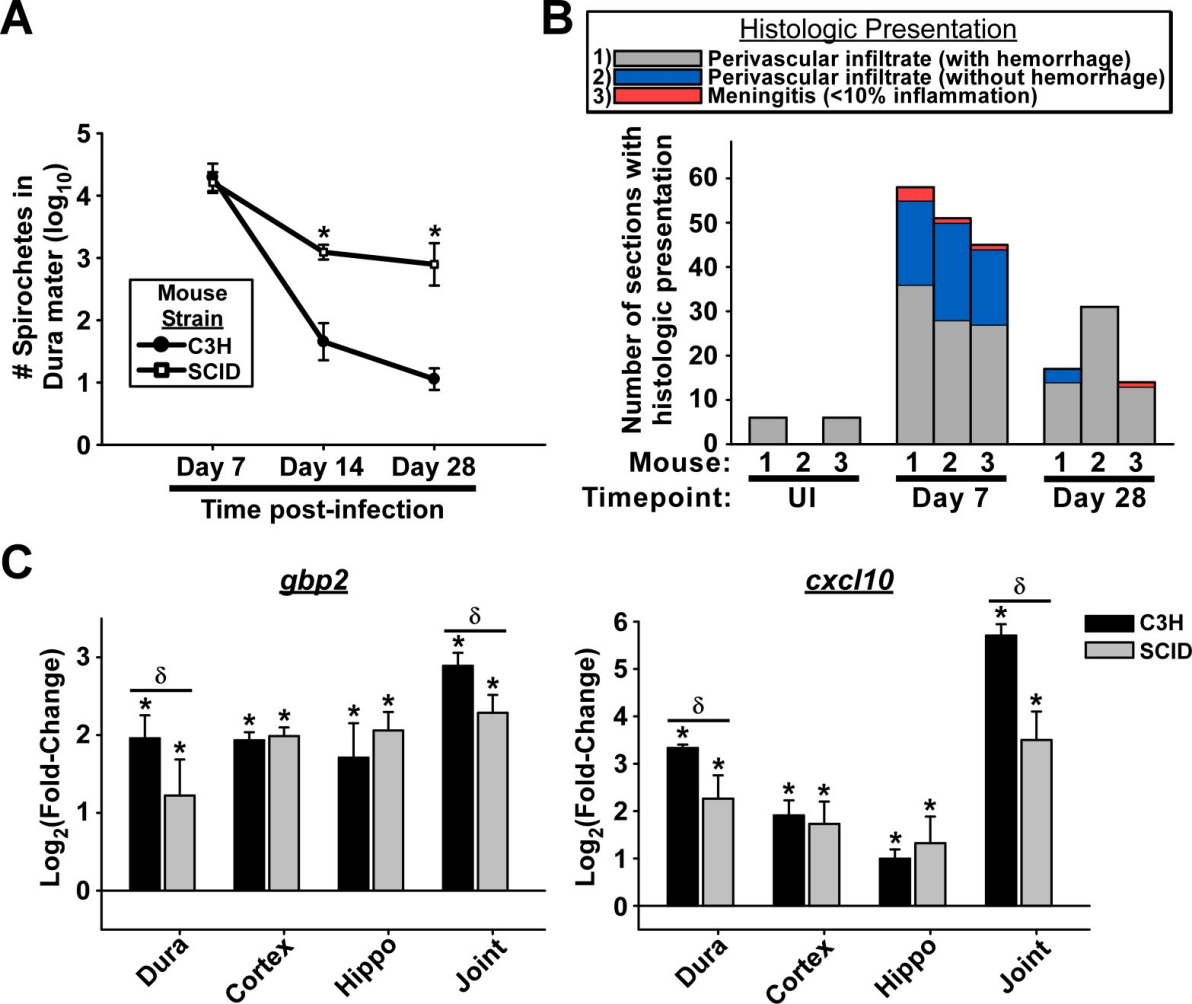
B cells and T cells are required for controlling spirochete burden during persistent infection, however are not required for leukocyte infiltration or upregulation of ISGs. **A.** Bacterial burden (log_10_ mean ± s.d.) in the dura mater of C3H vs SCID mice from day 7–28 post-infection (n = 3 per condition). Spirochetes were counted in isolated dura mater by systematic counting using f-IHC, epifluorescence microscopy, and gridded coverslips, as described in Materials and Methods and Fig 1B. Asterisks indicate statistical significance (p ≤ 0.001; α = 0.05) using Student’s t-test. **B.** Histopathology of dura mater from SCID mice at days 7–28 post-infection and uninfected controls (see detailed description in the legend of Fig 4). Stacked bargraph shows number of sections (out of 60) from each mouse with histologic presentations as shown in the legend. Timepoint of infection is shown for each mouse. **C.** qRT-PCR of indicated ISGs from dura mater (dura), cortex, hippocampus (hippo), and tibiotarsal joint (joint) of C3H and SCID mice at day 7 post-infection (n = 3). Bars are displayed as log_2_(fold-change) ± s.d. compared to uninfected control samples for each tissue as determined using the ΔΔCt method. Asterisks indicate statistical significance compared to uninfected controls (0.001 ≤ p ≤ 0.04; α = 0.05), whereas δ indicates differences between C3H and SCID mice (0.004 ≤ p ≤ 0.04; α = 0.05) using Student’s t-test.

Studies using immunodeficient SCID and *rag*-/- mice have clearly demonstrated a role for B cells and T cells in controlling *B*. *burgdorferi* burden in peripheral tissues during persistent infection; however, these cells are not required for early induction of ISGs (C3H-SCID) or the development of murine Lyme arthritis or carditis in disease susceptible strains (C3H-SCID, C3H *rag*-/-, BALB/c *rag*-/-) [19,21–23]. Similarly, Bb_297 burden was comparable in the dura mater of wild-type C3H and SCID mice at day 7 post-infection, whereas bacterial numbers were elevated in SCID mice at days 14 and 28 post-infection (Fig 10A), indicating a role for adaptive immunity in dura spirochete control during persistent infection.

Histopathologic analysis of the dura mater from infected SCID mice showed a similar temporal pattern of leukocyte infiltration as observed in wildtype mice in response to *B*. *burgdorferi* infection, with more prevalent perivascular leukocyte infiltrate at day 7 post-infection compared to similarly treated C3H mice (Figs 10B and 4A and S8). Although we did not attempt to identify specific differences in leukocyte subsets in the dura mater of SCID mice, these results are consistent with previous reports that murine Lyme arthritis and carditis characterized by leukocytic infiltrate occurs independent of the presence of B or T cells [21,22].

Induction of ISGs also occurred independently of T cells or B cells, as levels of both *gbp2* and *cxcl10* were elevated in infected SCID mice compared to uninfected controls in the dura, cortex, hippocampus, and tibiotarsal joints. Although T cells and B cells were not required for ISG induction above background levels, the magnitude of ISG response was modestly reduced in dura and joints of SCID mice compared to C3H mice, suggesting a role for T cells or B cells in supporting the induction of ISGs (Fig 10C).

Collectively, these data show that T cells and B cells are essential for pathogen control, but are not required for leukocytic infiltrate or IFN response in the CNS of mice during infection with *B*. *burgdorferi*, consistent with previous reports in peripheral tissues of these animals [19,21–23].

## Discussion

### The dura mater is a site of *Borrelia* colonization in mice during early and subacute infection

We report here for the first time, the kinetics of *B*. *burgdorferi* colonization of the meninges as well as the CNS host response to infection in a tractable laboratory animal model of Lyme disease. Following infection of mice by either tick transmission or subdermal needle inoculation in the dorsal thoracic skin, *B*. *burgdorferi* readily colonized the extravascular dura mater, reaching peak burdens of over 10^4^ bacteria in the examined calvaria-assocated dura at 7 days post-infection.

Previous studies have shown that both *B*. *burgdorferi* dissemination and disease severity can vary in laboratory mice depending on the site of inoculation [48,49]. Likewise, we found that the early peak of spirochetes in the dura mater was dependent on the proximity of the site of inoculation. Despite the ability to readily culture spirochetes from the blood of all mice at day 7 post-infection, dura spirochetes were rarely detected in mice inoculated in the footpad, and spirochete burdens were dramatically reduced in mice inoculated in the dorsal lumbar skin compared to thoracic skin. Thus, the presence of spirochetes in the blood at day 7 post-infection is not sufficient for the early peak of colonization in the dura mater. Dissemination of *B*. *burgdorferi* in mammals occurs by both hematogenous and non-hematogenous routes, such as through the lymphatic system or direct spread through tissues [50]. Although we did not quantify the kinetics of blood dissemination following different routes of infection and therefore cannot rule out differences in hematogenous dissemination, our results suggest that non-hematogenous spread may contribute to increased early colonization of the dura mater in mice. This increase was restricted to early infection, however, as spirochete burdens in the dura mater were comparable by 28 days post-infection regardless of inoculation site.

Epidemiological evidence suggest that some Lyme disease *Borrelia* are more likely to cause neurological complications than others [51]. We did not find evidence of differences in CNS colonization between *Borrelia* species or intraspecies isolates in our model. The similar distribution of spirochetes in the dura mater between isolates Bb_B31 and Bb_297 suggests that ribosomal spacer type (RST) and OspC type are not determinants of dura colonization among *B*. *burgdorferi* s.s. isolates [52]. We were also able to readily culture both North American and European *B*. *burgdorferi* s.l. species from the dura mater of perfused infected animals, further demonstrating the generalized nature of dura colonization in mice for Lyme disease spirochetes.

Bb_297 burdens in the dura mater peaked at day 7 post-infection, and by days 14–28 were quickly reduced to low levels similar to that reported during late disseminated infection [33]. Intriguingly, this early peak and clearance of spirochetes more closely resembles that reported in blood, rather than other peripheral tissues where burdens peak between 2–3 weeks post-infection and remain relatively higher throughout infection [7,53–55]. This rapid reduction of spirochete numbers in the dura mater suggests immune pressures similar to those seen in the blood, and was at least in part due to an adaptive immune response as burdens were significantly higher in SCID mice beyond day 7 post-infection. It has been proposed that the ability of *B*. *burgdorferi* to persist in peripheral tissues in the face of a strong anti-*Borrelia* immune response is due to tissue-specific protective niches established by host ECM molecules including decorin with high affinity binding to the *B*. *burgdorferi* surface [56]. Given the high levels of decorin, fibronectin, and multiple collagen types found in the dura mater, it is perhaps surprising that this tissue does not serve as such a strong protective niche. Nonetheless, the ability of *B*. *burgdorferi* to persist at low levels in this tissue for at least 75 days post-infection compared to undetectable levels in the blood during persistent infection may be due to bacterial interactions with these host proteins.

We did not detect spirochetes in the brains of infected animals by culture, qRT-PCR, or fluorescence microscopy, supporting the view that brain colonization in these animals is rare [32].

### *Borrelia* colonization of the dura mater is associated with a localized inflammatory response

The dura mater possesses fenestrated blood vessels, lymphatic drainage, and a high density of resident immune cells including dendritic cells (DC), mast cells (MC), innate lymphocytes (ILCs), meningeal macrophages, T cells, and B cells capable of supporting a robust immune response [57]. Indeed, colonization of the dura mater by Bb_297 was associated with a leukocytic influx of T cells and myeloid cells including monocytes/macrophages, concurrent with a decrease in B cells. Moreover, widespread changes in gene expression including an inflammatory cytokine and chemokine profile consistent with *B*. *burgdorferi*-stimulated TLR activation and NF-κB signaling was observed in the dura mater that was sustained up to 8 weeks post-infection. In addition to this inflammatory cytokine response, a transient IFN response was demonstrated that was not dependent on T cells or B cells, consistent with previous studies in *B*. *burgdorferi*-infected mice [14,23]. An early IFN response has been shown to be associated with spirochete dissemination, and contributes to murine arthritis pathology as well as affecting the cellular composition of lymph nodes in infected mice [14,23,29,58–60].

It is tempting to speculate that this robust inflammatory response in the dura mater could prime the animal for an inflammatory environment in the leptomeninges and the brain. Although we did not examine the CSF response in this study, a number of genes upregulated in the dura mater of infected mice have been reported to be elevated in the CSF of Lyme neuroborreliosis patients including cytokines/chemokines as well as the matrix metalloproteinases MMP3 and MMP9 [61–63]. Of these, the B cell chemokine Cxcl13 has become of particular interest due to its strong association with Lyme neuroborreliosis compared to healthy controls and patients with other neuroinflammatory diseases [61,64]. Co-culture experiments have demonstrated that *B*. *burgdorferi* induces Cxcl13 production in human dendritic cells and murine synovial cells [26]-[65]. A recent study reported that Type I IFN induced Cxcl13 production in platelet-derived growth factor receptor α (PDGFRα)+ pulmonary fibroblasts, but not hematopoietic cells, epithelial cells, or endothelial cells in response to Influenza A virus leading to recruitment of B cells [66]. The presence of dendritic cells and fibroblasts combined with the induction of a strong IFN response in the dura mater implicates either of these cell types as potential sources of the increase in dura *cxcl13* transcript in mice infected with *B*. *burgdorferi*. Despite the strong induction of *cxcl13* in the dura mater, we did not observe evidence of the presence of ectopic germinal centers by histology, and flow cytometry and gene expression profiles suggest a decrease in B cell numbers at day 7 post-infection. This may be due to temporal limitations of our experimental design, and induction of *cxcl13* during early infection may in fact be a leading indicator of increased B cell numbers in the dura mater during persistent infection.

An interesting finding of this study was the increase in leukocyte influx associated with vascular hemorrhage in histologic sections of the dura mater, cerebral meninges, ventricles, and choroid plexus of mice infected with Bb_297. Hemorrhagic strokes (e.g. intracranial hemorrhage, sub-arachnoid hemorrhage, and intraventricular hemorrhage) can all result in blood-brain-barrier (BBB) permeability, brain edema, and neuroinflammation characterized by an influx of leukocytes including neutrophils, monocytes, and T cells into the brain as well as activation of resident microglia within hours to days following hemorrhage [67]. That we did not identify edema or leukocytic infiltrates in the brain parenchyma is noteworthy, and suggest that the observed hemorrhage in *B*. *burgdorferi* infected mice may have occurred during the process of euthanasia. Further studies on the effects of *B*. *burgdorferi* infection on the vasculature of the CNS in live animals are currently underway to address this hypothesis. Although the intracranial hemorrhaging reported here may have happened peri-mortem rather than in live infected animals, the fact that this phenomenon was not seen in uninfected animals suggests that the intracranial vasculature is compromised during *B*. *burgdorferi* infection, which could prime the animal for potentially pathologic consequences including increased susceptibility to CNS inflammation. Although rare, cerebrovascular manifestations have been associated with Lyme disease in humans [68].

We observed decreased expression of several ECM components important for vascular integrity in the dura mater at day 7 post-infection including BM-associated collagens, laminins, and other structural proteins in addition to increased expression of matrix metalloprotease genes *mmp3* and *mmp9* [69,70]. Additionally, we found decreased expression of genes encoding gap and adherens junction proteins including connexins, ZO-1, and VE-cadherin. This finding in conjunction with altered expression of ECM components and MMPs provide a potential mechanism for the vascular changes seen in infected mice [67,69,70]. The *B*. *burgdorferi*-induced breakdown in vascular integrity in the dura mater may explain the rapid accumulation of spirochetes and immune cells during early infection. Further effects on the vasculature in other tissues or barrier functions in the meninges that could contribute to CNS pathology are yet to be determined.

### Mice infected with *B*. *burgdorferi* exhibit a sterile immune response in the brain parenchyma during early infection

Perhaps our most intriguing finding was evidence of an immune response in the cerebral cortex and hippocampus, despite the absence of spirochete detection in the parenchyma by molecular, microscopic, or bacterial culture methods. Specifically, we observed primarily upregulation of ISGs and genes involved in antigen presentation by both MHCI and MHCII in both cortex and hippocampus of infected mice, as well as increased expression of the gene coding for CD11c in the cerebral cortex. To our knowledge, this is the first description of a tissue-specific sterile immune response to *B*. *burgdorferi* infection in mice. Previous work has demonstrated that soluble components of cell-free *B*. *burgdorferi* spent medium (including nucleic acids and yet-unidentified non nucleic acid ligands) can govern IRF3-dependent, IFN-responsive gene transcription [71]. Additionally, mouse models of peripheral Type I IFN injection and other models of peripheral sterile inflammation have been reported to stimulate induction of ISGs and microglial activation in the brain parenchyma [72–75]. Given that the gene expression profile in the brain of *B*. *burgdorferi*-infected mice is largely ISGs and markers of cell activation (i.e. MHC-associated genes), it is possible that the sterile brain inflammation in our model could be caused by soluble host or bacterial factors that perpetuate inflammatory signals to deeper CNS tissues where spirochetes are absent. Indeed, Lyme disease patients with encephalopathy and headache in the absence of confirmed neuroborreliosis (i.e. normal CSF clinical test results) share similar profiles of elevated serum cytokines compared to patients with non-Lyme encephalopathy, suggesting that there may be a shared systemic inflammatory etiology that contributes to these neurological manifestations [76].

The downstream effects of the observed gene expression changes in the brains of *B*. *burgdorferi*-infected mice is not known; however, it is noteworthy that both Type I and Type II IFN can contribute to inflammatory pathology in murine models of Lyme arthritis and carditis [23,29,30,60]. In mice, models of peripheral sterile inflammation can be associated with physiologic and behavioral symptoms of sickness in addition to induction of gene expression changes including ISG induction in the brain [73–75,77–81]. Additionally, peripherally administered Type I IFNs can cause a number of side effects in humans, such as depression-associated symptoms including fatigue, insomnia, irritability, loss of appetite, and cognitive changes [82–85]. It is also notable that altered MHC II expression in the brain has been linked to many neurodegenerative diseases in humans and mouse models, and increased CD11c has been demonstrated in transcriptomic studies from both total cortex and acutely isolated microglia in several mouse models of neurodegenerative disease [86]. Although the potential pathologic consequences of the observed changes in the brain parenchyma are not clear in our model, our findings demonstrate that the brain is not simply a naïve bystander during *B*. *burgdorferi* infection of laboratory mice.

## Conclusion

Overall, the findings reported in this study are significant, as the lack of a tractable animal model has hindered our understanding of host-pathogen interactions in the CNS during *B*. *burgdorferi* infection. Our results provide insight into potential mechanisms of CNS pathologies associated with Lyme disease, and describe a model system that will allow for future studies evaluating the bacterial, host, and environmental factors that can contribute to the severity of CNS involvement during *B*. *burgdorferi* infection. Such studies are critical for the development and implementation of novel prophylactic and therapeutic interventions for this important disease.

## Materials and methods

### Ethics statement

All biohazard and animal experiments were carried out in accordance with approved protocols from the University of North Dakota Institutional Biosafety Committee (protocol number IBC-201204-006) and the Animal Care and Use Committee (Animal Welfare assurance number A3917-01), respectively.

### Bacterial strains and culture conditions

Low passage *B*. *burgdorferi* isolate 297 strain CE162 (Bb_297) and GFP expressing isogenic mutant Bb914 (Bb_297-GFP) were obtained as gifts from Melissa Caimano and Justin Radolf [87,88]. *B*. *burgdorferi* strain B31 clone MI-16 was obtained as a gift from Brian Stevenson [89]. *B*. *mayonii* strain MN14-1539 was obtained as a gift from Jeanine Peterson [37]. *B*. *garinii* strain Ip90 was obtained as a gift from Troy Bankhead [36]. To confirm the presence of plasmids that were required for infectivity, plasmid content for each strain of *B*. *burgdorferi* was analyzed by multiplex PCR with primers specific for regions unique to each plasmid, as previously described [90,91]. Prior to all animal infections, spirochetes were cultured to mid-log phase in BSK-II medium at 37°C, 5% CO2, and quantified by dark field microscopy using a Petroff-Hausser chamber [92].

### Animal infections

Immunocompetent C3HeB/FeJ (C3H; stock # 000658) and immunodeficient C3SnSmn.Cg-*Prkdc*^*scid*^/J (SCID; stock # 001131) were purchased from The Jackson Laboratory (Sacramento, CA). For all experiments, 6–8 week-old female mice were used, and housed in groups unless otherwise noted.

For infections by needle inoculation, animals were placed under anesthesia using isoflurane inhalation, followed by subdermal inoculation with 100 uL of BSK-II medium containing the indicated inoculation dose and strain of *Borrelia* (infected animals), or medium alone (uninfected controls). Inoculation site included the dorsal thoracic midline, dorsal lumbar midline, or footpad, as indicated.

For infections by tick transmission, larval *Ixodes scapularis* ticks were obtained from BEI Resources (Manassas, VA). Four weeks post-infection by needle inoculation, mice were anesthetized with isoflurane and briefly placed in a tube with naïve larval ticks. To facilitate long-term tick attachment, the mice were placed in individual restrainers overnight. The following day, mice were released and placed on a wire rack suspended over water and provided with food and water ad libitum. Replete ticks were collected daily, stored at 23°C with 12-hour light/dark cycles, and allowed to molt to nymphs. For infection of mice by tick transmission, infected nymphs were applied to naïve mice and allowed to feed as above (10 ticks per mouse placed on dorsal thoracic region). Post transmission feeding, the number of recovered replete nymphal ticks ranged from 4–6 per mouse. Recovered ticks were crushed, and cultured in BSK medium to confirm infection. All ticks were culture positive for spirochetes post-transmission feeding with the exception of one.

Infections were confirmed in mice by collecting ~80 μL blood from the saphenous vein at day 7 post-infection and cultured in BSK-II supplemented with 20-μg ml−1 phosphomycin, 50-μg ml−1 rifampicin, and 2.5-μg ml−1 amphotericin-B. Ear tissue was also isolated and cultured at time of sacrifice of all animals. Dark-field microscopy was used to confirm the presence of viable spirochetes for each cultured blood/tissue sample.

### Immunohistochemistry, epifluorescence, and confocal imaging

Samples were stained for imaging of spirochetes, T cells, and endothelial vessels as previously described [33,93]. Briefly, each sample was post-fixed in 4% paraformaldehyde (PFA) for 24h at 4°C. Samples were permeabilized in 0.1% Triton X-100, washed 3 times, and serum-blocked in 2.5% goat serum/PBS containing 1:100 dilution of Fc block (CAT # 553142; BD Biosciences, San Jose CA). For *B*. *burgdorferi* staining, each sample was incubated in 1:100 dilution of rat anti-mouse unconjugated monoclonal anti-CD31 IgG (BD; CAT # 550274), and 1:50 dilution biotinylated rabbit anti-*B*. *burgdorferi* polyclonal IgG (Invitrogen; CAT# PA1-73007; Thermo-Fisher Scientific, Waltham, MA) at 4°C overnight. On the following day, the samples were washed, and stained with 1:100 dilution of Alexa 555 goat anti-rat polyclonal IgG (Invitrogen; CAT # A-21434), and 1:200 dilution of Alexa 488 streptavidin (Invitrogen; CAT # S11223) for 1 hour at room temperature, covered from light. For CD3 staining, each sample was primary stained using 1:200 dilution of rabbit unconjugated polyclonal anti-CD3 IgG (CAT # ab5690; Abcam, Cambridge, MA), or an equivalent concentration of rabbit unconjugated anti-mouse polyclonal IgG as an isotype control (Abcam; CAT # ab37415). Secondary staining was performed using 1:600 dilution of goat Alexa 488 polyclonal anti-rabbit IgG (Abcam; CAT # ab150081). CD31 staining was performed as described above. For all samples, secondary antibody staining alone was done as a negative control for non-specific signal, and spleen sections were stained as positive control for CD3 binding as previously described [33]. After antibody staining, samples were incubated in PBS containing 1uM TOPRO-3 nuclear stain for 10 minutes, followed by 2 more washes. Each sample was placed onto a positively charged glass slide and mounted using VECTASHIELD antifade mounting medium (CAT # H-1200; Vector Labs, Burlingame, CA) and gridded coverslip (Electron Microscopy Sciences, Hatfield, PA).

Spirochetes and CD3+ cells stained with Alexa 488 secondary antibody were identified from separate samples by epifluorescence based on morphology and positive signal in the FITC channel using an Olympus BX-50 (Olympus; Center Valley, PA) at 200x magnification as previously described [33]. Bacteria were quantified by manual counting in a blinded fashion, with the identity of the sample being unknown to the researcher during counting. Samples with more than 1000 positive events were counted by systematic random sampling using the gridded coverslips, counting every tenth grid square [94]. No significant bias in the distribution of spirochetes were observed with regards to distance from dural sinuses that would impact the accuracy of systematic counts. Images were acquired from representative samples above using a Zeiss LSM 510 confocal microscope (Ziess-US; White Plains, NY), and data was collected using Olympus Cell Sens software followed by image processing using Fiji [33,95].

### Intravital microscopy

Two photon microscopy was performed on ex vivo dura mater using a modified protocol as described in [96]. Skullcaps with attached dura were isolated by craniotomy from mice infected with Bb_297-GFP at day 7 post-infection. Freshly isolated skullcaps were inverted and immobilized to a 100mm X 15mm petri dish, and the exposed dura was covered in PBS to provide a medium for immersion of the objective lens. Imaging was immediately performed in real time using an Olympus FV1000 MPE basic upright multiphoton laser scanning microscope equipped with a tunable MaiTai DS IR laser (690–1040 nm range).

Images were acquired using an Olympus XLPLN 25X, 1.05NA water immersion lens with zoom set to 3.0 and the IR laser tuned to 910 nm. Emission wavelengths of 420–460 nm (violet; second harmonic generation) and 495–540 nm (green: GFP) were used to image connective tissue and the bacteria respectively. Images were acquired using continuous frame capture at 5.43 seconds per frame for 40 frames per image sequence. Image resolution was 165 nm/pixel. Image processing, analysis, and video construction was done using Fiji [95].

### Histopathology

Calvaria with attached dura were isolated by craniotomy followed by immediate fixation in 4% PFA for 24h at 4°C. Fixed samples were decalcified in 0.4M EDTA in PBS for 48h at room temperature followed by serial dehydration in 10%/20%/30% sucrose, frozen in Tissue-Tek OCT (CAT # 4583; Sakura Finetek USA, Torrence, CA), and cut on a cryostat in 6μm coronal sections. Representative sections from each sample (every 35^th^ 6μm section) were stained with haematoxylin and eosin for evaluation by light microscopy. Sections were scored on an increasing scale of 0–3 as follows: No inflammatory cell infiltrate (score = 0); Perivascular infiltrates associated with hemorrhage (score = 1); Perivascular infiltrates without hemorrhage (score = 2); Meningitis (<10% meningitis; score = 3). All histopathology scores were determined by an ACVP board certified veterinary pathologist who was masked to the identity of the samples.

Coronal sections (10 μm) of brains isolated from the same animals were processed in a similar manner without decalcification step, and scored using criteria identical to dura samples.

Representative images of dura mater and brain sections were obtained using an Epredia Pannoramic MIDI II slide scanner. Images are composites generated using the extended focus z-stack function (0.2μM step size X 5 steps per image).

### Flow cytometry

Single cells were dissociated by incubating dura isolated from transcardially perfused mice with 500 units/ml type 4 collagenase (CAT # LS004188; Worthington Biochemicals, Lakewood, NJ) and 100 μg/ml DNaseI (Worthington; CAT # LS006333) in PBS (45 min, 37°C). Digestion reactions were quenched in PBS + 5% FBS, and the supernatant and remaining dura tissue were triturated on ice with a 200 μl pipette tip and strained through a 100 μm sieve. Cell suspensions were washed in PBS, followed by staining with Zombie Aqua viability dye (1:200; CAT# 423102; Biolegend; San Diego, CA). Cells were pretreated prior to antibody staining with TruStain FcX (1:25; Biolegend CAT# 156604) in PBS + 5% FBS. The following antibodies were purchased from Biolegend, and used to label cells in PBS + 5% FBS: PerCP/Cyanine5.5 anti-mouse CD45 (1:40; CAT# 103132), FITC anti-mouse CD3ε (1:50; CAT# 100306), Brilliant Violet 605 anti-mouse CD19 (1:50; CAT# 115540), PE anti-mouse/human CD11b (1:40; CAT# 101208), PE/Cyanine7 anti-mouse Ly-6G (1:100; CAT# 127618), APC/Cyanine7 anti-mouse F4/80 (1:20; CAT# 123118), True-Stain Monocyte Blocker (1:20; CAT# 426102). Samples were run on a BD FACSymphony. Gating strategy is shown in S1 Fig. Flow cytometric data were analyzed using FCS Express (De Novo Software).

### RNA isolation

For gene expression analysis, mice were anesthetized using isofluorane and perfused transcardially with 4 mL PBS followed by 4 mL RNAlater (Invitrogen; CAT # AM7020) using a peristaltic pump at a flow rate of 0.8mL/min. Dura, heart, and joint tissues were isolated as previously described and immediately snap-frozen in liquid nitrogen prior to storage at −80°C. Brains were removed and stored in 4 mL RNAlater at 4°C for < 1 week prior to processing. A 2mm thick coronal slice of each brain was manually dissected under cold RNAlater to isolate cortex and hippocampus from surrounding regions including removing the meninges and corpus callosum. Isolated cortex and hippocampus were snap-frozen in liquid nitrogen and stored at -80°C.

Tissues from 3 separate animals were combined per sample prior to RNA extraction, and represented a single biological replicate. Frozen tissues were ground under liquid nitrogen, added to 1 ml of pre-warmed (65°C) TRIzol reagent (Invitrogen; CAT # 15596026), and frozen at −80°C overnight. Trizol suspensions were thawed at room temperature, and RNA was isolated using the Direct-zol RNA minikit (CAT # R2052; Zymo Research, Irvine CA) according to the manufacturer's instructions. RNA concentration was determined by a Qubit 2.0 fluorometer (Life Technologies; Carlsbad, CA), and RNA integrity was verified by microfluidic-based capillary electrophoresis with an Agilent 2100 Bioanalyzer (RNA integrity number [RIN] ≥ 8.5 for all samples).

### RNA sequencing

cDNA libraries were prepared from 250ng of purified input RNA using the NEBNext Ultra II kit (CAT# E7770S) with Poly(A) mRNA Magnetic Isolation Module (CAT# E7490S) and index PCR primers (CAT #s E7335, E7500) (New England Biolabs; Ipswich MA) according to the manufacturer's instructions. Library concentration was assessed with a BioTek Gen5 Wellplate reader with the Quant-iT PicoGreen dsDNA Assay kit (Thermo Fisher; CAT # P11496), and analyzed on the Bioanalyzer to ensure appropriate size distributions and rule out adaptor contamination.

The indexed cDNA libraries were pooled and 150 bp paired-end reads were sequenced on two lanes using the Illumina HiSeq 4000 (Novogene; San Diego, CA). Demultiplexed fastq files from the two sequencing runs were combined for each sample, and read quality confirmed using FASTQC v0.11.2 prior to analysis.

For the analysis of transcriptome sequencing (RNA-seq) data, adapters were removed from the sequencing reads by Trimmomatic v0.32 [97]. Reads were aligned to the murine genome (mm10) using STAR v2.7.1a with 2-pass mapping [98]. Fragments were assigned to genes using featureCounts v1.6.4 [99]. Differential expression analysis was performed using DESeq2 v1.24.0 [100]. Genes were considered to be differentially expressed in infected samples compared to control samples at a false discovery rate (padj) of ≤0.05, basemean>20, fold-change >1.5.

Functional analysis (gene ontology) of upregulated or downregulated genes was performed using clusterProfiler v3.12.0 [101]. KEGG pathway enrichment analysis from all DEGs was performed using Signaling Pathway Impact Analysis (SPIA) v2.36.0 with Bonferroni correction and a significance threshold of pGFWER ≤ 0.05 [102].

### Quantitative reverse transcriptase PCR (qRT-PCR)

cDNA was generated from 500ng of purified RNA using Superscript IV First-Strand Synthesis System with included RnaseH treatment (Invitrogen; Cat # 18091050). Quantitative PCR for the *B*. *burgdorferi flaB* gene was performed in 20μL reactions with 12.5ng cDNA, gene-specific primers, and internal fluorescent probes using Bio-Rad SsoAdvanced™ Universal probes Supermix (CAT # 1725281; Bio-Rad, Hercules, CA) as previously described [103]. Absolute copy numbers were interpolated for each sample in triplicate using standard curves. Host gene expression was determined with individual PCR primer sets (*gapdh*, CAT# QT01658692; *tnfa*, CAT# QT00104006; *gbp2*, CAT# QT00106050; *cxcl10*, CAT# QT00093436; Qiagen USA, Germantown, MD) using Bio-Rad SsoAdvanced Universal SYBR Green Supermix (Bio-Rad; CAT #1725274). Relative changes in gene expression were compared between infected and control animals using the 2^-ΔΔCt^ method with *gapdh* as a housekeeping control.

### Statistical analysis

Statistical analysis of RNA sequencing data is described above in the “RNA sequencing” subsection of Materials and Methods. Statistical tests used to compare means for all other experiments were performed using Sigmaplot v11.0 (Systat Software; San Jose, CA) and are described in the relevant Fig legends. For microscopy counting experiments, sample sizes were calculated a priori using G*Power v 3.1.9.7 using the following input parameters: α = 0.05; Power = 0.8; sample N allocation ration = 1; effect size = 5. For qRT-PCR confirmation of RNA-seq results, n = 4 was used to maintain consistency with RNA-seq experiment.

### Data visualization

Data generated from DNA and RNA sequencing analyses were visualized with R v.3.3.0 (https://www.R-project.org/) using the following packages: clusterProfiler v3.12.0 for GO term analysis and cnet plots [101]; Pathview for KEGG pathway DEG visualization [104]; ggplot2 for volcano plots [105]; NMF v0.21.0 for heatmaps [106]. All other plots were generated using Sigmaplot v11.0 (Systat Software).

## Supporting information

S1 Movie *B*. *burgdorferi* in the dura mater are extravascular and motile. Multiphoton image series of dura mater from C3H mouse after infection with GFP-Bb_297 for 7 days. *B*. *burgdorferi* is shown in green; second harmonics shown in blue. Two minute movie. Imaging parameters: Wavelength = 910 nm, pixel resolution = 135.
(AVI)

S1 Table Differential expression gene list in the dura mater. (XLSX)

S2 Table Differential expression gene list in the cortex. (XLSX)

S3 Table Differential expression gene list in the hippocampus. (XLSX)

S1 Fig Flow cytometric gating strategy for the identification of leukocyte subsets. **A.** Live events were defined based on forward scatter height vs. area (singlets), forward scatter vs. side scatter areas (cells of interest), and intensity of amine-reactive dye (live cells). Average number of live events ± s.d. = 5524 ± 2643 across all dura samples. Example plots shown are from representative control spleen sample. **B.** Gating strategy for identification of leukocyte subsets. Example plots shown are from uninfected control spleen (F4/80 fluorescence minus one control), and dura mater samples from uninfected and 7-day infected mice as indicated. Leukocyte subtypes were defined as: T cells: CD45+CD3+; B cells: CD45+CD19+; Monocyte/Macrophage-enriched: CD45+CD3-CD19-CD11b+Ly6G-; Granulocyte-enriched: CD45+CD3-CD19-CD11b+Ly6G+.
(TIF)

S2 Fig Gene expression changes are consistent across biological replicates. **A.** Principal component analysis of all samples used in this study. Samples are color-coded by treatment (uninfected (UI) vs 7 day Bb_297 infected (Bb)); while tissues are denoted by symbol shape as shown in the legend. **B.** Hierarchical clustering of individual samples from RNA-seq data. Columns represent individual samples, while rows represent individual DEGs. Colors represent Euclidean distances from the regularized log-transformed counts (rlog) generated using DESeq2 for all DEGs. Separate heatmaps are displayed for each tissue as indicated in the titles. Treatment groups (uninfected, UI1-4; 7 day infected, Bb1-4) cluster together for each tissue, and show similar profiles of upregulated/downregulated genes in response to infection.
(TIF)

S3 Fig Genes upregulated in the dura mater and brain parenchyma represent different gene ontologies. Top five most enriched molecular function gene ontology (GO) terms from upregulated genes in the dura mater **(A)**, cortex **(B)**, hippocampus **(C)**, and genes commonly upregulated in all three tissues **(D)**. Numbers to the right of horizontal bars show the number of upregulated DEGs associated with each term. Bar size represents significance of enrichment (-log(padj)).
(TIF)

S4 Fig Toll-like receptor signaling is increased in the dura mater in response *to B*. *burgdorferi* infection. **A.** Summary of Signaling Pathway Impact Analysis (SPIA) for TLR signaling (mmu04620). Table shows number of DEGs for each tissue (dura, cortex, hippocampus) within the pathway, as well as the activation status of the pathway (n.s. = not significant). pGFWER represents the false discovery rate after Bonferroni correction. **B.** DEGs were mapped onto the KEGG pathway as rendered using Pathview [104]. Pathway gene products (such as receptors, adaptors and enzyme proteins) are represented as rectangles, with interaction shown as arrows. Rectangles are color coded by log_2_(fold-change) from RNA-seq datasets (infect vs. uninfected), with the left one-third of the rectangle representing DEG status in the dura mater, the center representing DEG status in the cortex, and the right one-third representing DEG status in the hippocampus. Color scale is shown in the legend. Upregulation of most TLR signaling genes is restricted to the dura mater following infection.
(TIF)

S5 Fig NF-κB signaling is increased in the dura mater in response *to B*. *burgdorferi* infection. **A.** Summary of Signaling Pathway Impact Analysis (SPIA) for NF-κB signaling (mmu04064). Table shows number of DEGs for each tissue (dura, cortex, hippocampus) within the pathway, as well as the activation status of the pathway (n.s. = not significant). pGFWER represents the false discovery rate after Bonferroni correction. **B.** DEGs were mapped onto the KEGG pathway as rendered using Pathview [104]. Pathway gene products (such as receptors, adaptors and enzyme proteins) are represented as rectangles, with interaction shown as arrows. Rectangles are color coded by log_2_(fold-change) from RNA-seq datasets (infect vs. uninfected), with the left one-third of the rectangle representing DEG status in the dura mater, the center representing DEG status in the cortex, and the right one-third representing DEG status in the hippocampus. Color scale is shown in the legend. Upregulation of most NF-κB signaling genes is restricted to the dura mater following infection.
(TIF)

S6 Fig Antigen processing and presentation is increased in the dura mater and brain parenchyma in response *to B*. *burgdorferi* infection. **A.** Summary of Signaling Pathway Impact Analysis (SPIA) for antigen processing and presentation (mmu04612). Table shows number of DEGs for each tissue (dura, cortex, hippocampus) within the pathway, as well as the activation status of the pathway (n.s. = not significant). pGFWER represents the false discovery rate after Bonferroni correction. **B.** DEGs were mapped onto the KEGG pathway as rendered using Pathview [104]. Pathway gene products (such as receptors, adaptors and enzyme proteins) are represented as rectangles, with interaction shown as arrows. Rectangles are color coded by log_2_(fold-change) from RNA-seq datasets (infect vs. uninfected), with the left one-third of the rectangle representing DEG status in the dura mater, the center representing DEG status in the cortex, and the right one-third representing DEG status in the hippocampus. Color scale is shown in the legend. Upregulation of most antigen processing/presentation genes is seen in all three tissues following infection.
(TIF)

S7 Fig T cell receptor signaling is increased in the dura mater in response *to B*. *burgdorferi* infection. **A.** Summary of Signaling Pathway Impact Analysis (SPIA) for T cell receptor signaling (mmu04660). Table shows number of DEGs for each tissue (dura, cortex, hippocampus) within the pathway, as well as the activation status of the pathway (n.s. = not significant). pGFWER represents the false discovery rate after Bonferroni correction. **B.** DEGs were mapped onto the KEGG pathway as rendered using Pathview [104]. Pathway gene products (such as receptors, adaptors and enzyme proteins) are represented as rectangles, with interaction shown as arrows. Rectangles are color coded by log_2_(fold-change) from RNA-seq datasets (infect vs. uninfected), with the left one-third of the rectangle representing DEG status in the dura mater, the center representing DEG status in the cortex, and the right one-third representing DEG status in the hippocampus. Color scale is shown in the legend. Upregulation of most T cell receptor signaling genes is restricted to the dura mater following infection.
(TIF)

S8 Fig Infection with *B*. *burgdorferi* leads to leukocyte infiltration in the dura mater in SCID mice. Representative images (**A-B**) of perivascular leukocyte infiltrate in coronal sections of dura mater isolated from SCID mice (400X magnification).
(TIF)

S9 Fig Full resolution image of Fig 4B (100X). (TIF)

S10 Fig Full resolution image of Fig 4B (400X). (TIF)

S11 Fig Full resolution image of Fig 4C (100X). (TIF)

S12 Fig Full resolution image of Fig 4C (400X). (TIF)

S13 Fig Full resolution image of Fig 5A (100X). (TIF)

S14 Fig Full resolution image of Fig 5B (100X). (TIF)

S15 Fig Full resolution image of Fig 5C (100X). (TIF)

S16 Fig Full resolution image of Fig 5D (100X). (TIF)

S17 Fig Full resolution image of Fig 5D (400X). (TIF)

S18 Fig Full resolution image of Fig 5E (100X). (TIF)

S19 Fig Full resolution image of Fig 5E (400X). (TIF)

S20 Fig Full resolution image of Fig 5F (100X). (TIF)

S21 Fig Full resolution image of Fig 5F (400X). (TIF)
