# Supplementary figures and images for: A murine model of Lyme disease demonstrates that Borrelia burgdorferi colonizes the dura mater and induces inflammation in the central nervous system
Source: PLoS Pathog. 2021 Feb 1;17(2):e1009256. doi: 10.1371/journal.ppat.1009256 (PMC7877756; doi:10.1371/journal.ppat.1009256)

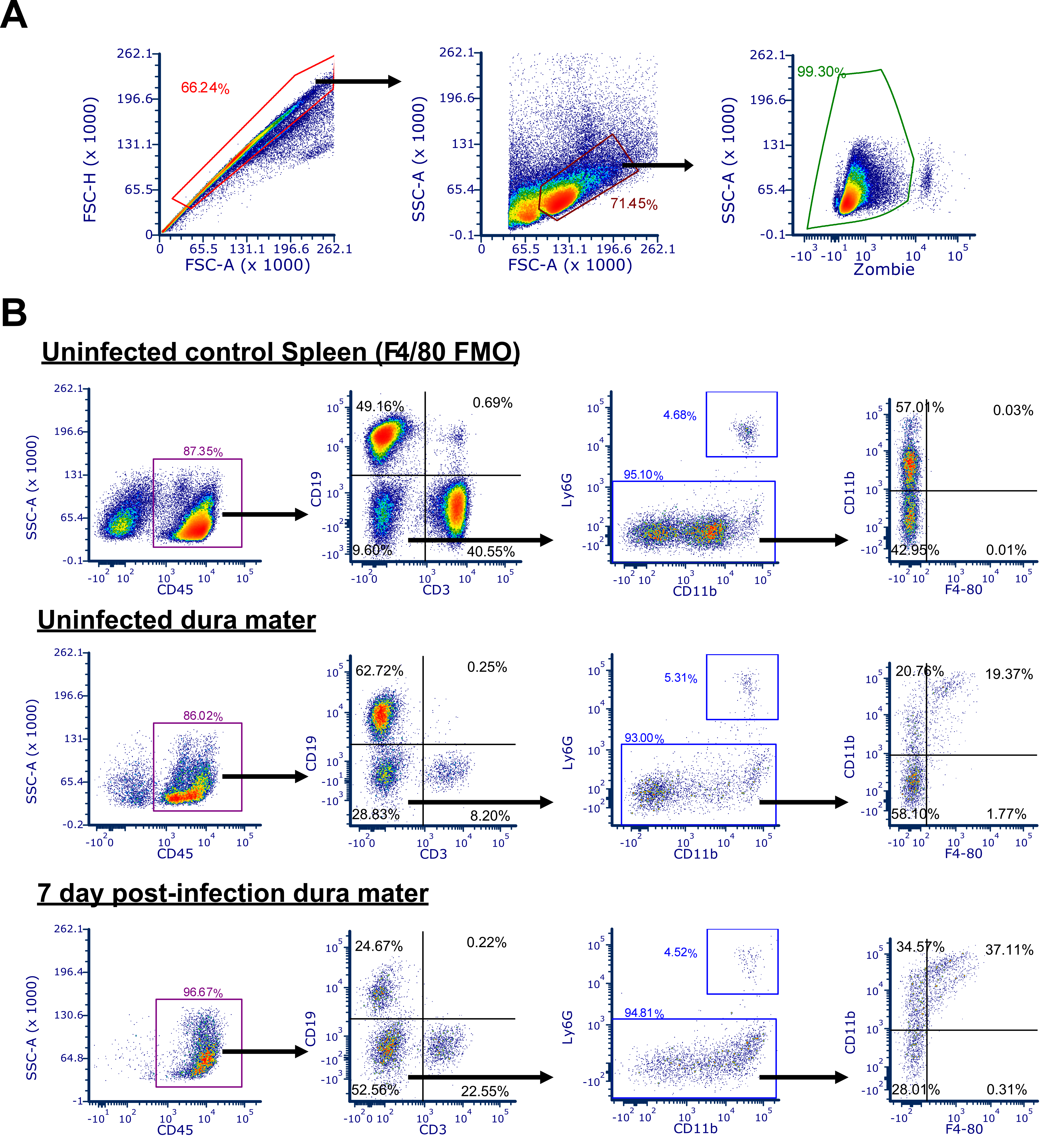

Supplement: S1 Fig — A. Live events were defined based on forward scatter height vs. area (singlets), forward scatter vs. side scatter areas (cells of interest), and intensity of amine-reactive dye (live cells). Average number of live events ± s.d. = 5524 ± 2643 across all dura samples. Example plots shown are from representative control spleen sample. B. Gating strategy for identification of leukocyte subsets. Example plots shown are from uninfected control spleen (F4/80 fluorescence minus one control), and dura mater samples from uninfected and 7-day infected mice as indicated. Leukocyte subtypes were defined as: T cells: CD45+CD3+; B cells: CD45+CD19+; Monocyte/Macrophage-enriched: CD45+CD3-CD19-CD11b+Ly6G-; Granulocyte-enriched: CD45+CD3-CD19-CD11b+Ly6G+. (TIF) [file ppat.1009256.s005.tif]

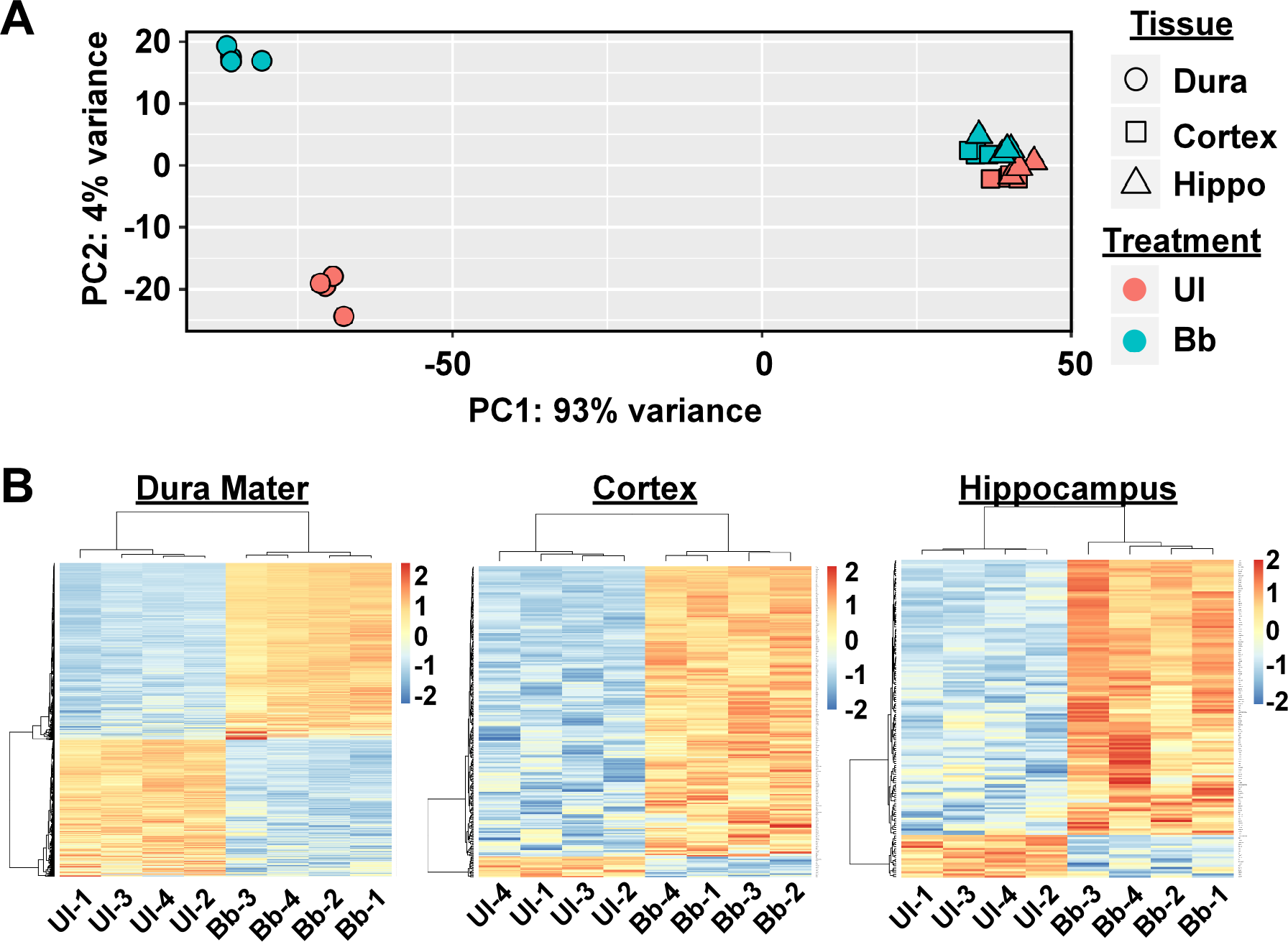

Supplement: S2 Fig — A. Principal component analysis of all samples used in this study. Samples are color-coded by treatment (uninfected (UI) vs 7 day Bb_297 infected (Bb)); while tissues are denoted by symbol shape as shown in the legend. B. Hierarchical clustering of individual samples from RNA-seq data. Columns represent individual samples, while rows represent individual DEGs. Colors represent Euclidean distances from the regularized log-transformed counts (rlog) generated using DESeq2 for all DEGs. Separate heatmaps are displayed for each tissue as indicated in the titles. Treatment groups (uninfected, UI1-4; 7 day infected, Bb1-4) cluster together for each tissue, and show similar profiles of upregulated/downregulated genes in response to infection. (TIF) [file ppat.1009256.s006.tif]

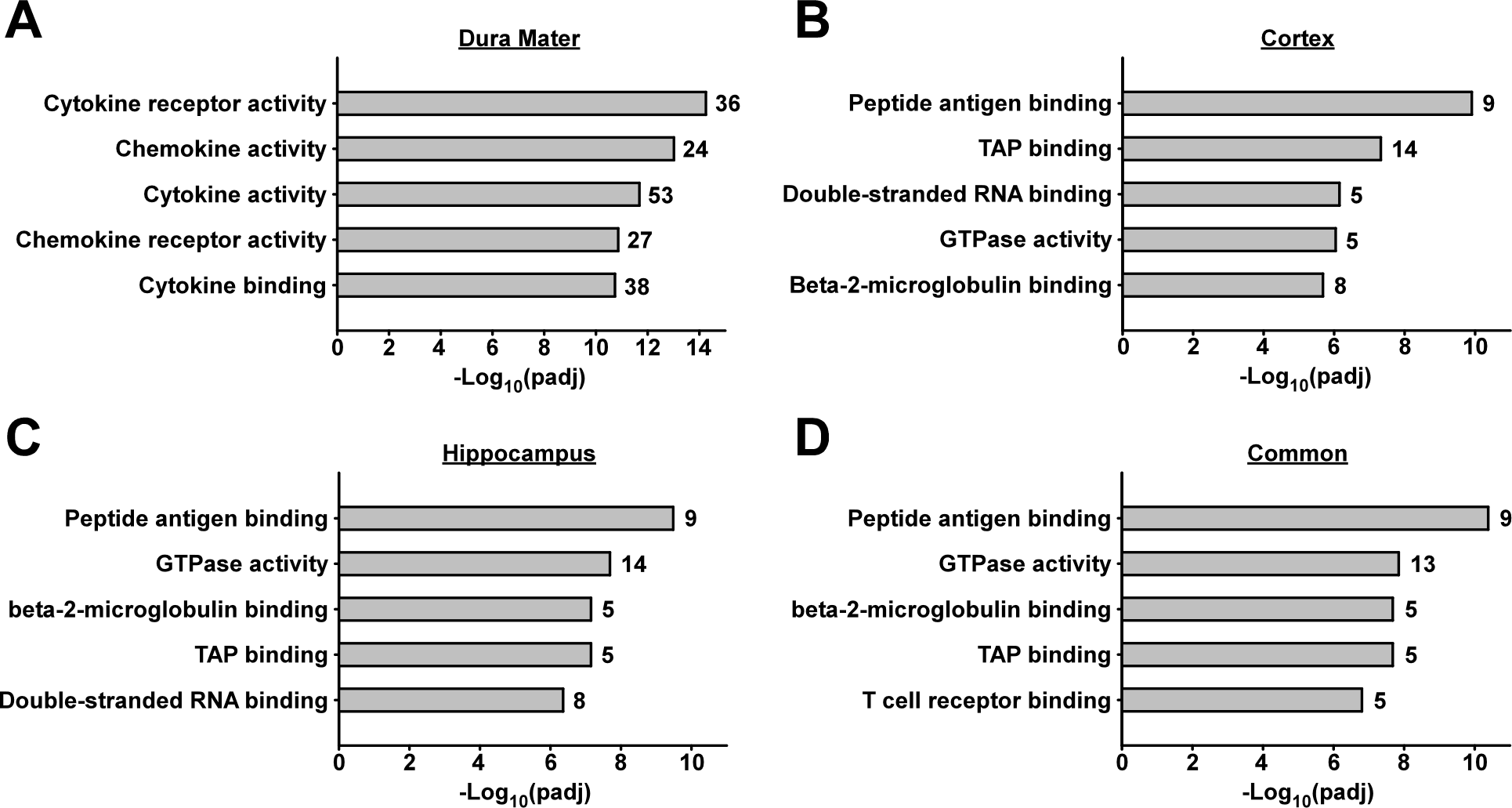

Supplement: S3 Fig — Top five most enriched molecular function gene ontology (GO) terms from upregulated genes in the dura mater (A), cortex (B), hippocampus (C), and genes commonly upregulated in all three tissues (D). Numbers to the right of horizontal bars show the number of upregulated DEGs associated with each term. Bar size represents significance of enrichment (-log(padj)). (TIF) [file ppat.1009256.s007.tif]

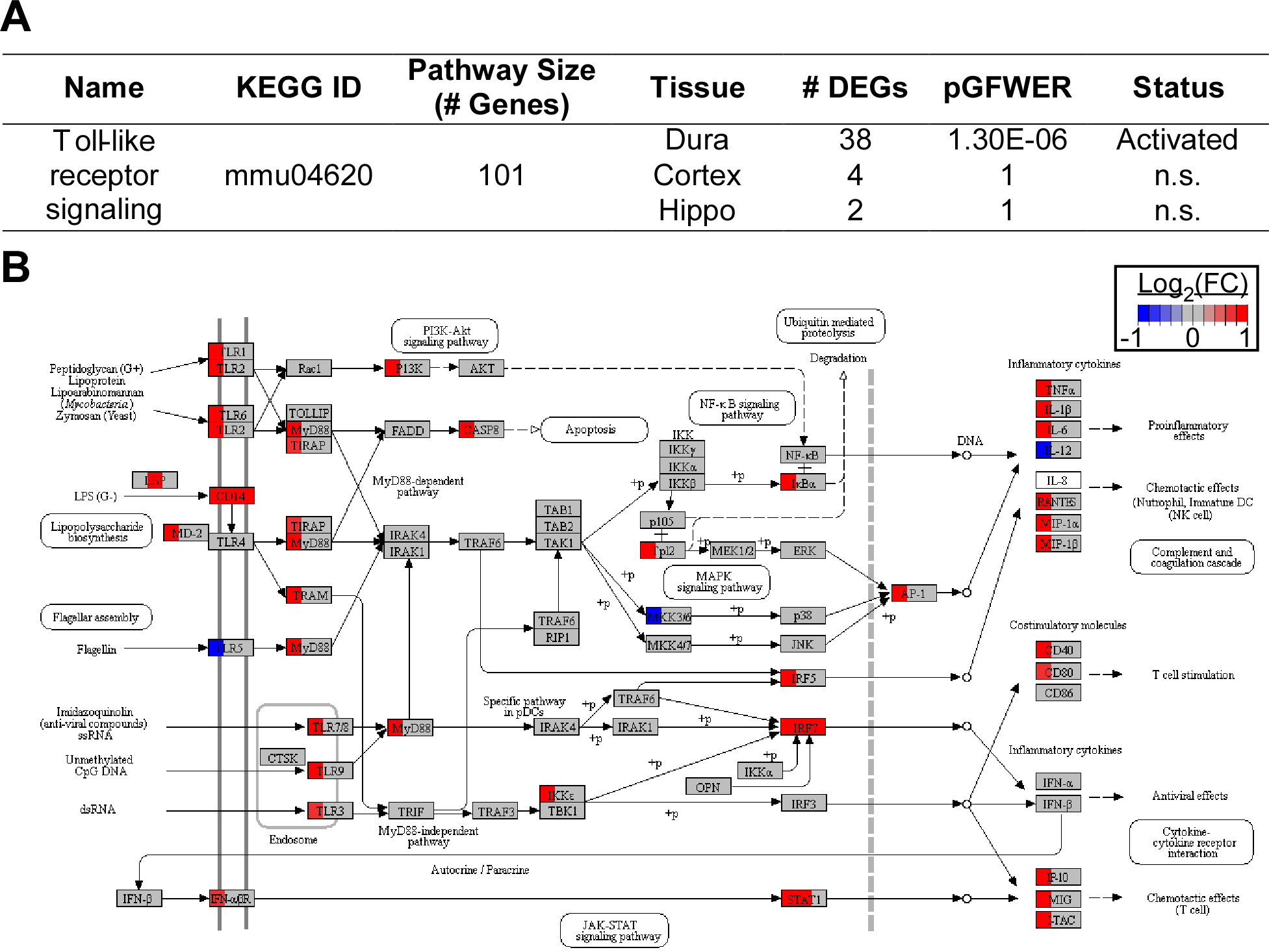

Supplement: S4 Fig — A. Summary of Signaling Pathway Impact Analysis (SPIA) for TLR signaling (mmu04620). Table shows number of DEGs for each tissue (dura, cortex, hippocampus) within the pathway, as well as the activation status of the pathway (n.s. = not significant). pGFWER represents the false discovery rate after Bonferroni correction. B. DEGs were mapped onto the KEGG pathway as rendered using Pathview [104]. Pathway gene products (such as receptors, adaptors and enzyme proteins) are represented as rectangles, with interaction shown as arrows. Rectangles are color coded by log2(fold-change) from RNA-seq datasets (infect vs. uninfected), with the left one-third of the rectangle representing DEG status in the dura mater, the center representing DEG status in the cortex, and the right one-third representing DEG status in the hippocampus. Color scale is shown in the legend. Upregulation of most TLR signaling genes is restricted to the dura mater following infection. (TIF) [file ppat.1009256.s008.tif]

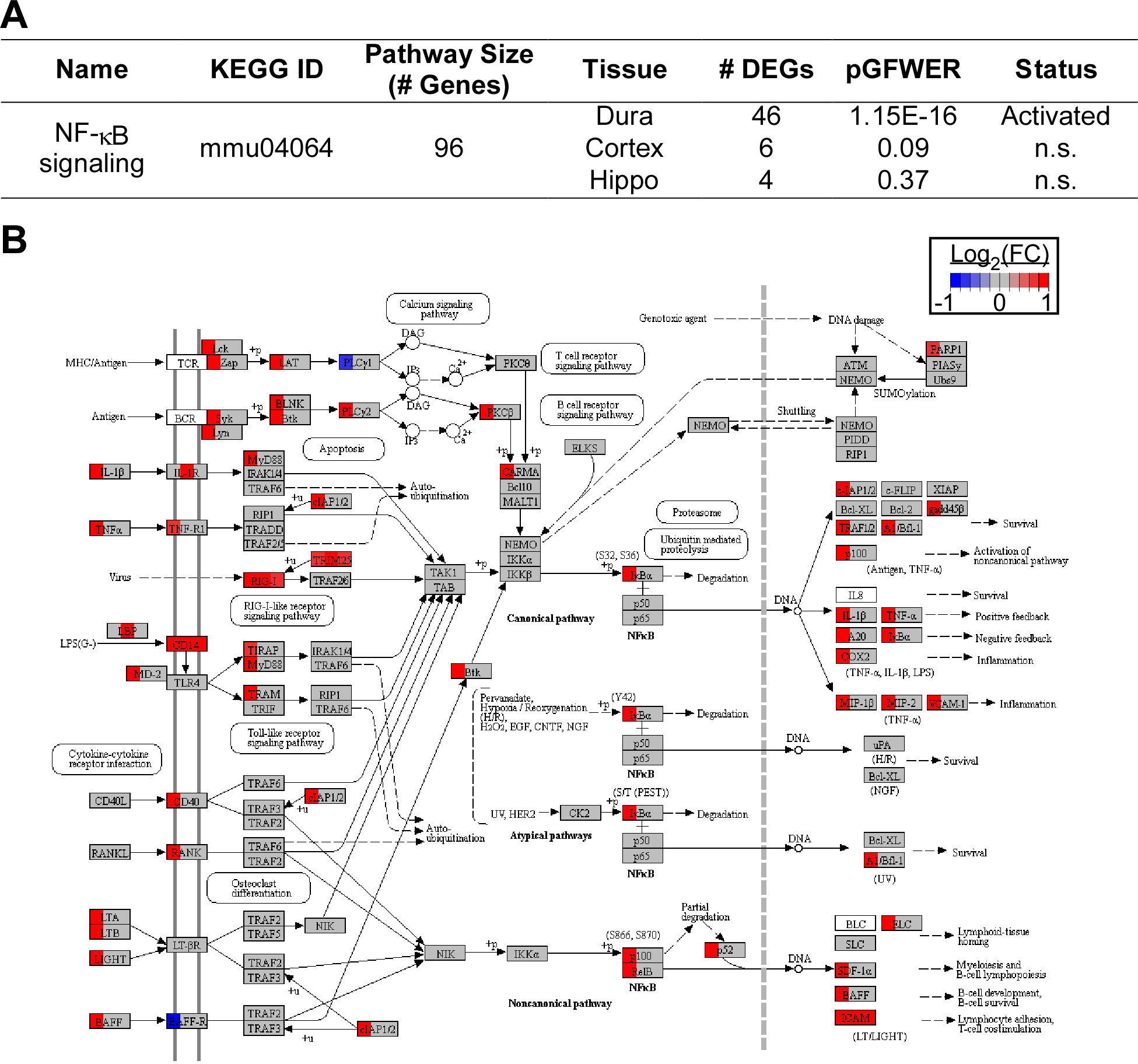

Supplement: S5 Fig — A. Summary of Signaling Pathway Impact Analysis (SPIA) for NF-κB signaling (mmu04064). Table shows number of DEGs for each tissue (dura, cortex, hippocampus) within the pathway, as well as the activation status of the pathway (n.s. = not significant). pGFWER represents the false discovery rate after Bonferroni correction. B. DEGs were mapped onto the KEGG pathway as rendered using Pathview [104]. Pathway gene products (such as receptors, adaptors and enzyme proteins) are represented as rectangles, with interaction shown as arrows. Rectangles are color coded by log2(fold-change) from RNA-seq datasets (infect vs. uninfected), with the left one-third of the rectangle representing DEG status in the dura mater, the center representing DEG status in the cortex, and the right one-third representing DEG status in the hippocampus. Color scale is shown in the legend. Upregulation of most NF-κB signaling genes is restricted to the dura mater following infection. (TIF) [file ppat.1009256.s009.tif]

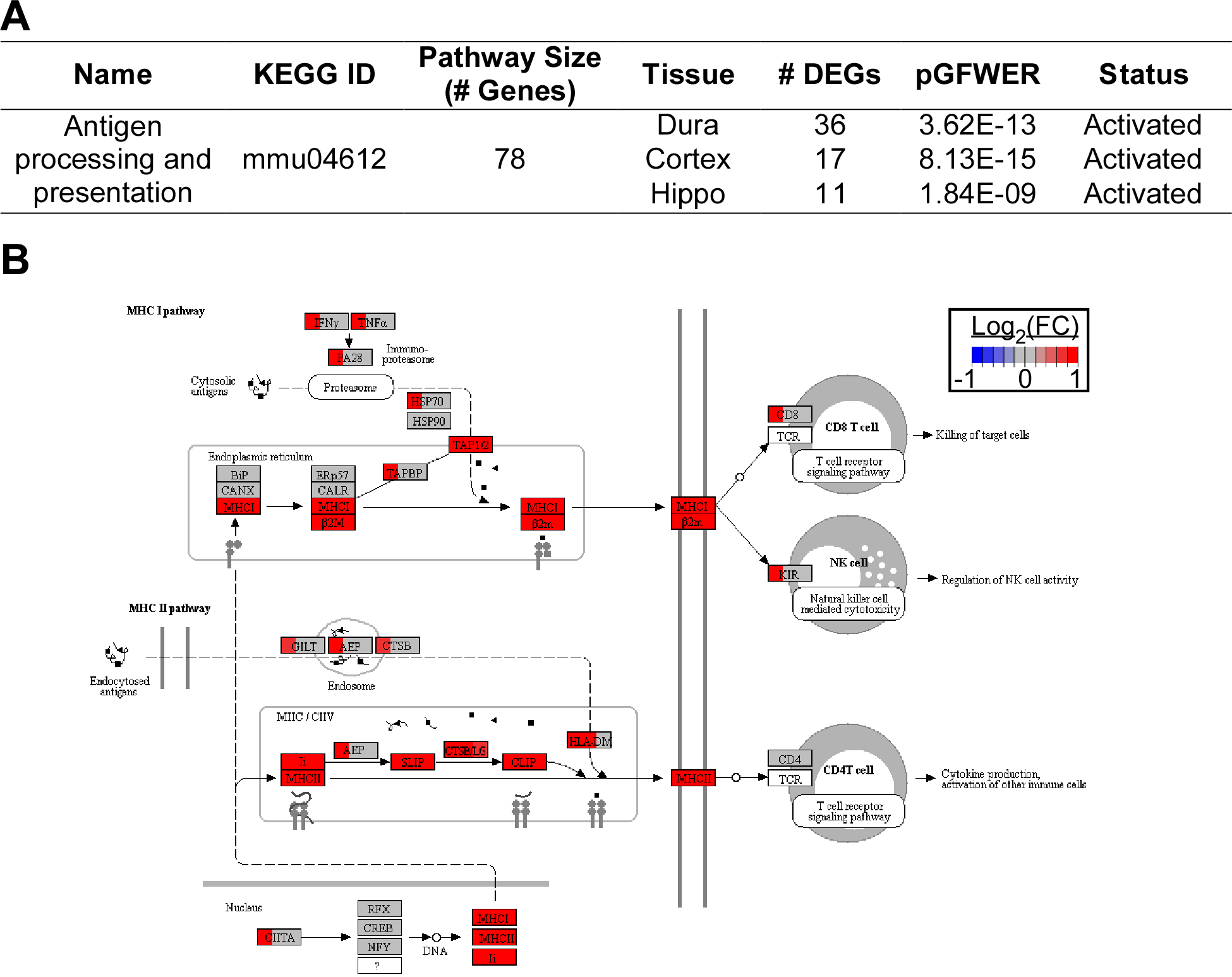

Supplement: S6 Fig — A. Summary of Signaling Pathway Impact Analysis (SPIA) for antigen processing and presentation (mmu04612). Table shows number of DEGs for each tissue (dura, cortex, hippocampus) within the pathway, as well as the activation status of the pathway (n.s. = not significant). pGFWER represents the false discovery rate after Bonferroni correction. B. DEGs were mapped onto the KEGG pathway as rendered using Pathview [104]. Pathway gene products (such as receptors, adaptors and enzyme proteins) are represented as rectangles, with interaction shown as arrows. Rectangles are color coded by log2(fold-change) from RNA-seq datasets (infect vs. uninfected), with the left one-third of the rectangle representing DEG status in the dura mater, the center representing DEG status in the cortex, and the right one-third representing DEG status in the hippocampus. Color scale is shown in the legend. Upregulation of most antigen processing/presentation genes is seen in all three tissues following infection. (TIF) [file ppat.1009256.s010.tif]

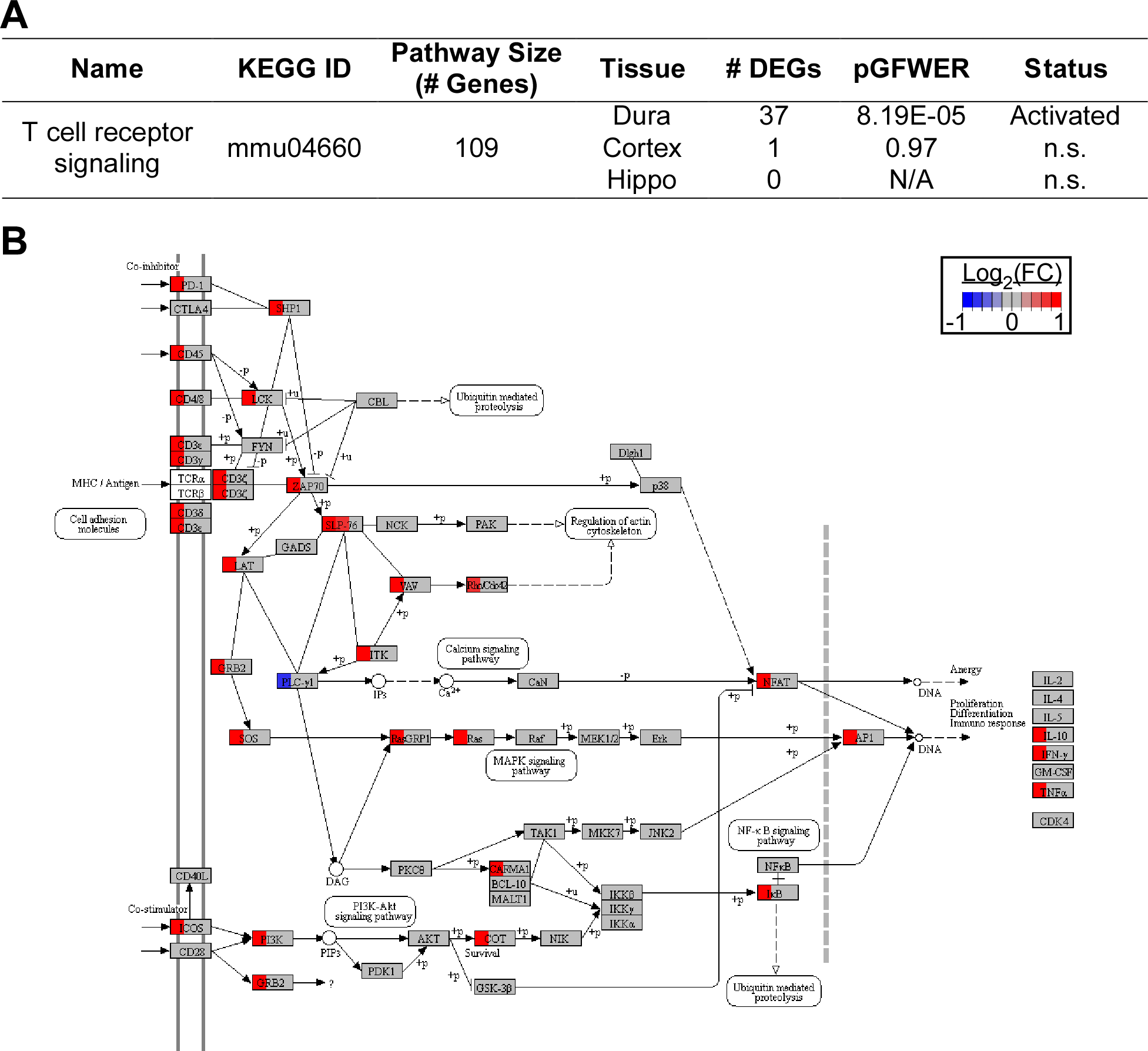

Supplement: S7 Fig — A. Summary of Signaling Pathway Impact Analysis (SPIA) for T cell receptor signaling (mmu04660). Table shows number of DEGs for each tissue (dura, cortex, hippocampus) within the pathway, as well as the activation status of the pathway (n.s. = not significant). pGFWER represents the false discovery rate after Bonferroni correction. B. DEGs were mapped onto the KEGG pathway as rendered using Pathview [104]. Pathway gene products (such as receptors, adaptors and enzyme proteins) are represented as rectangles, with interaction shown as arrows. Rectangles are color coded by log2(fold-change) from RNA-seq datasets (infect vs. uninfected), with the left one-third of the rectangle representing DEG status in the dura mater, the center representing DEG status in the cortex, and the right one-third representing DEG status in the hippocampus. Color scale is shown in the legend. Upregulation of most T cell receptor signaling genes is restricted to the dura mater following infection. (TIF) [file ppat.1009256.s011.tif]

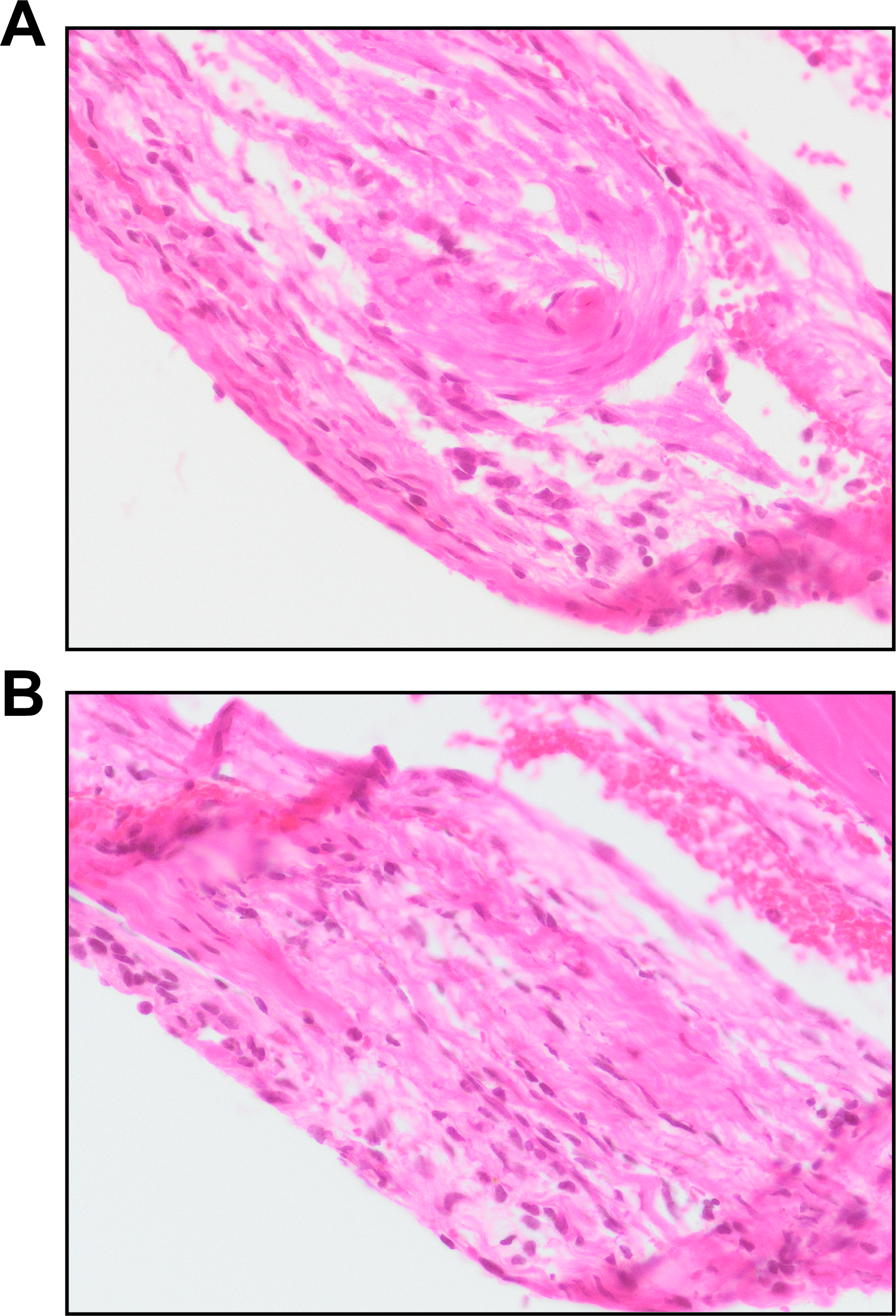

Supplement: S8 Fig — Representative images (A-B) of perivascular leukocyte infiltrate in coronal sections of dura mater isolated from SCID mice (400X magnification). (TIF) [file ppat.1009256.s012.tif]

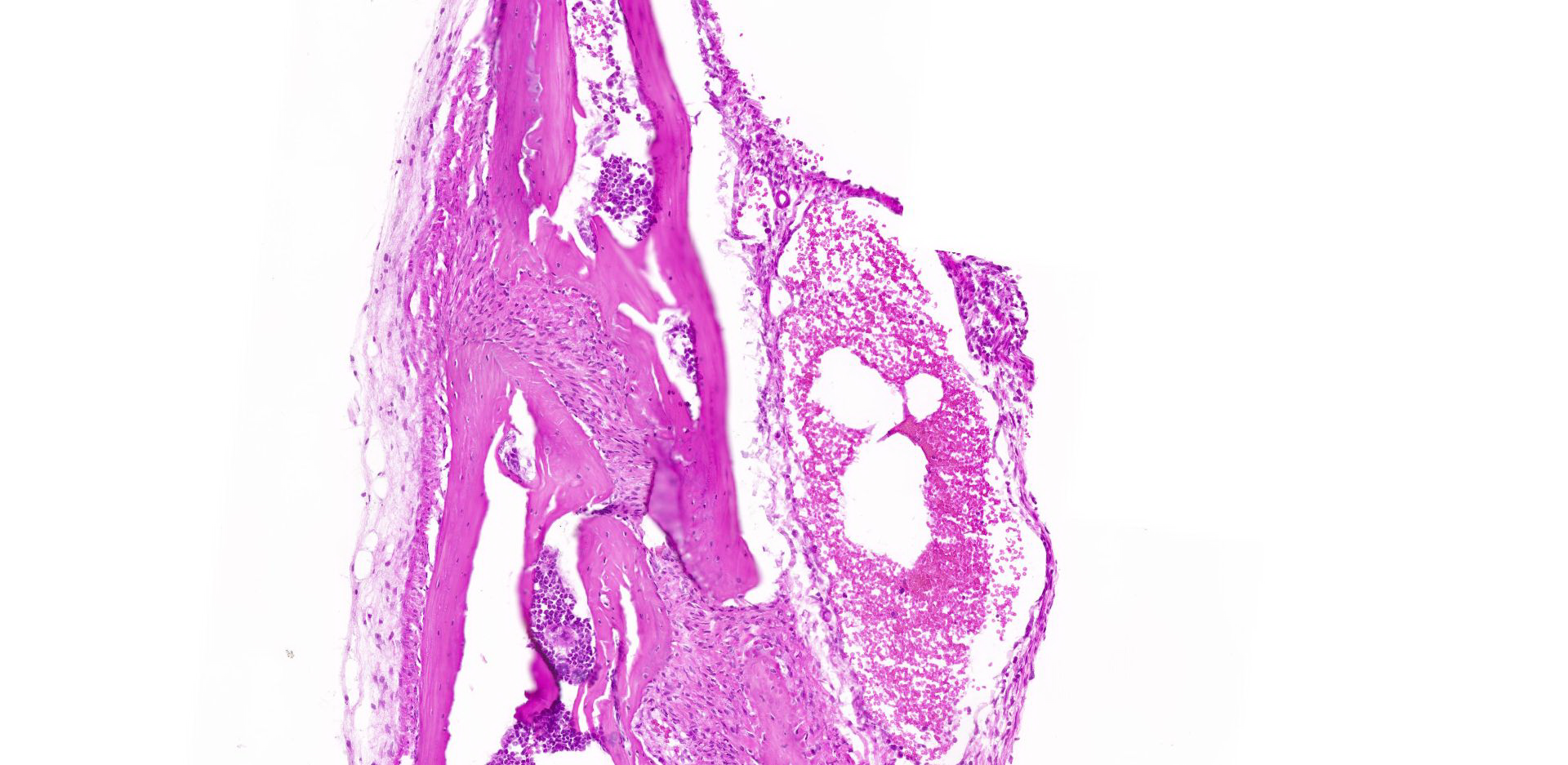

Supplement: S9 Fig — (TIF) [file ppat.1009256.s013.tif]

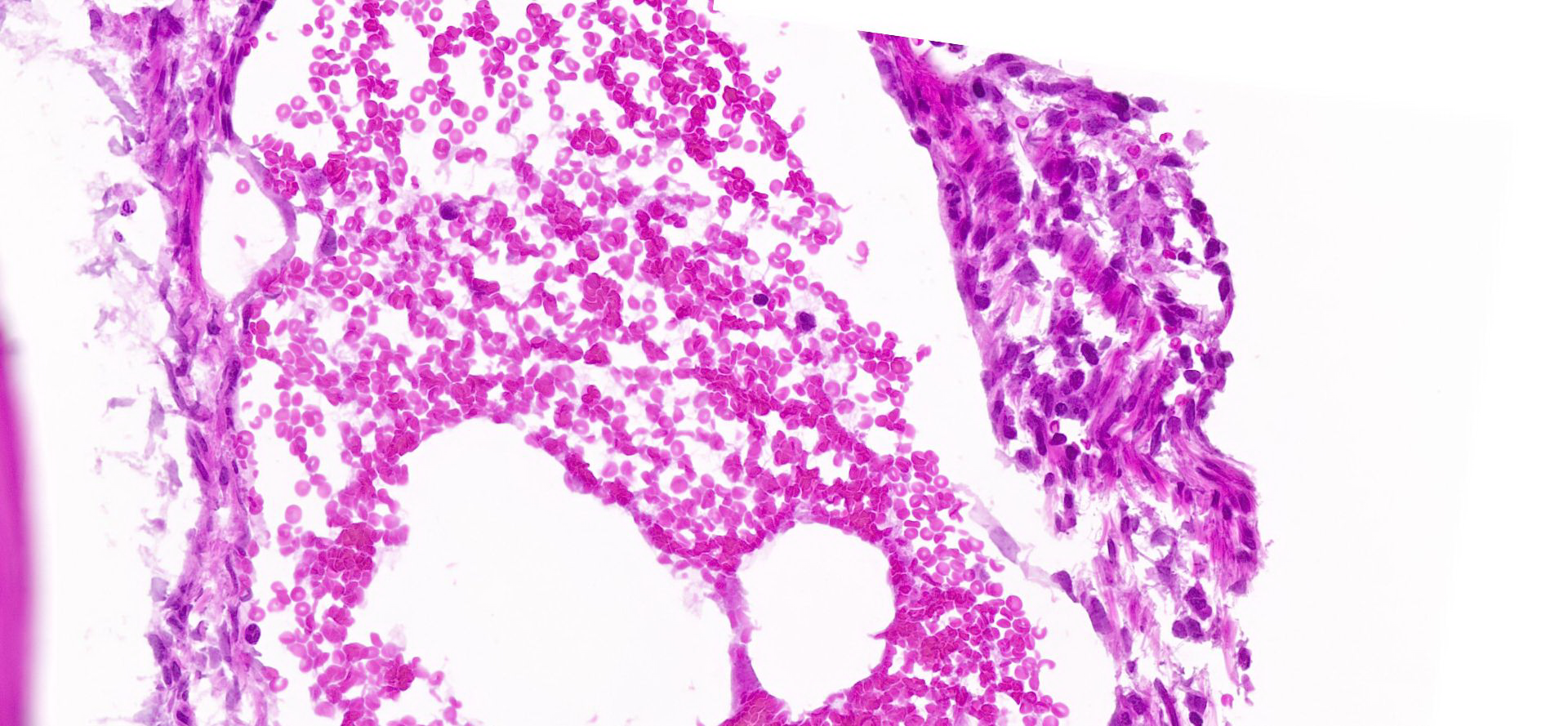

Supplement: S10 Fig — (TIF) [file ppat.1009256.s014.tif]

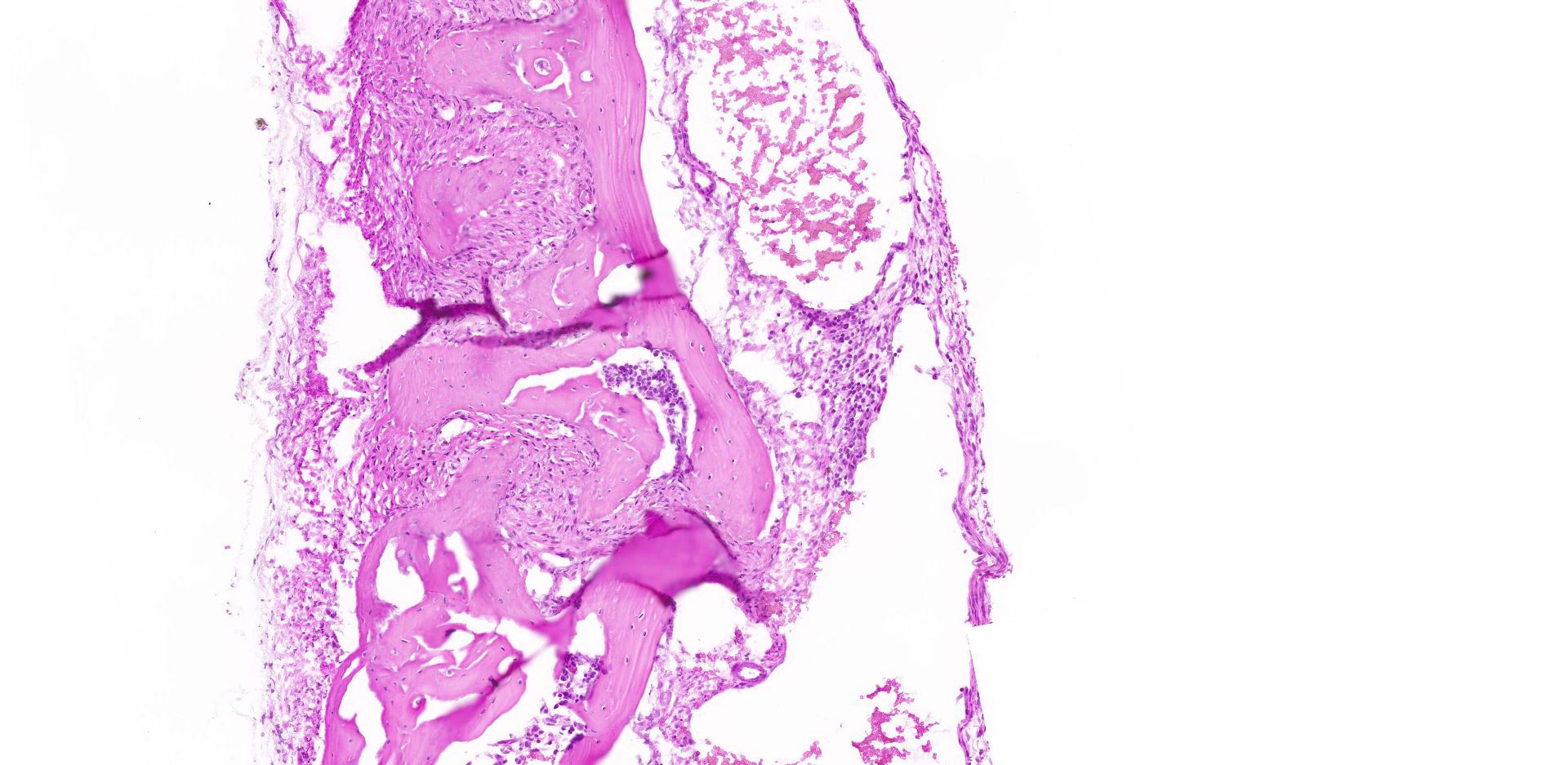

Supplement: S11 Fig — (TIF) [file ppat.1009256.s015.tif]

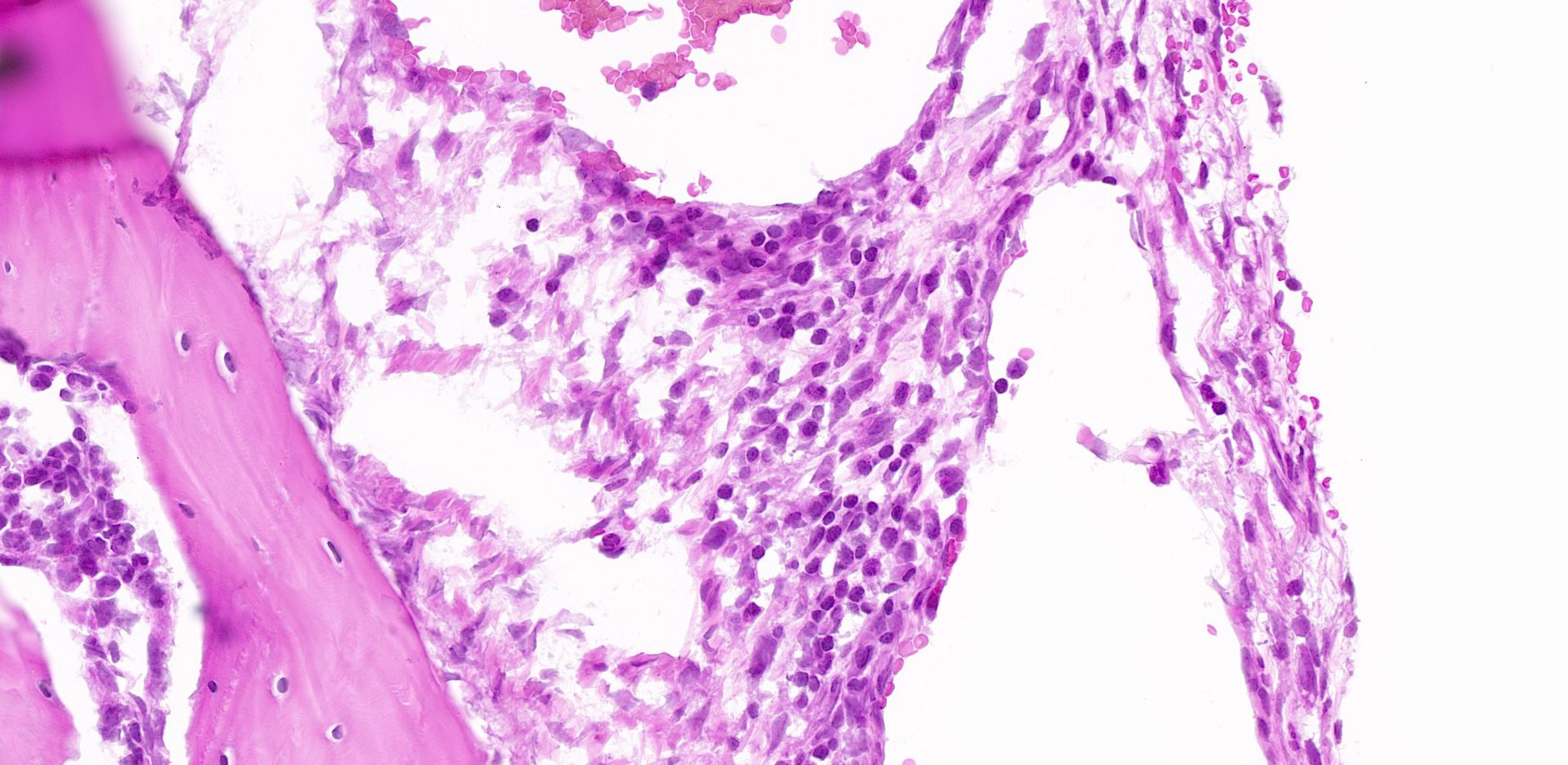

Supplement: S12 Fig — (TIF) [file ppat.1009256.s016.tif]

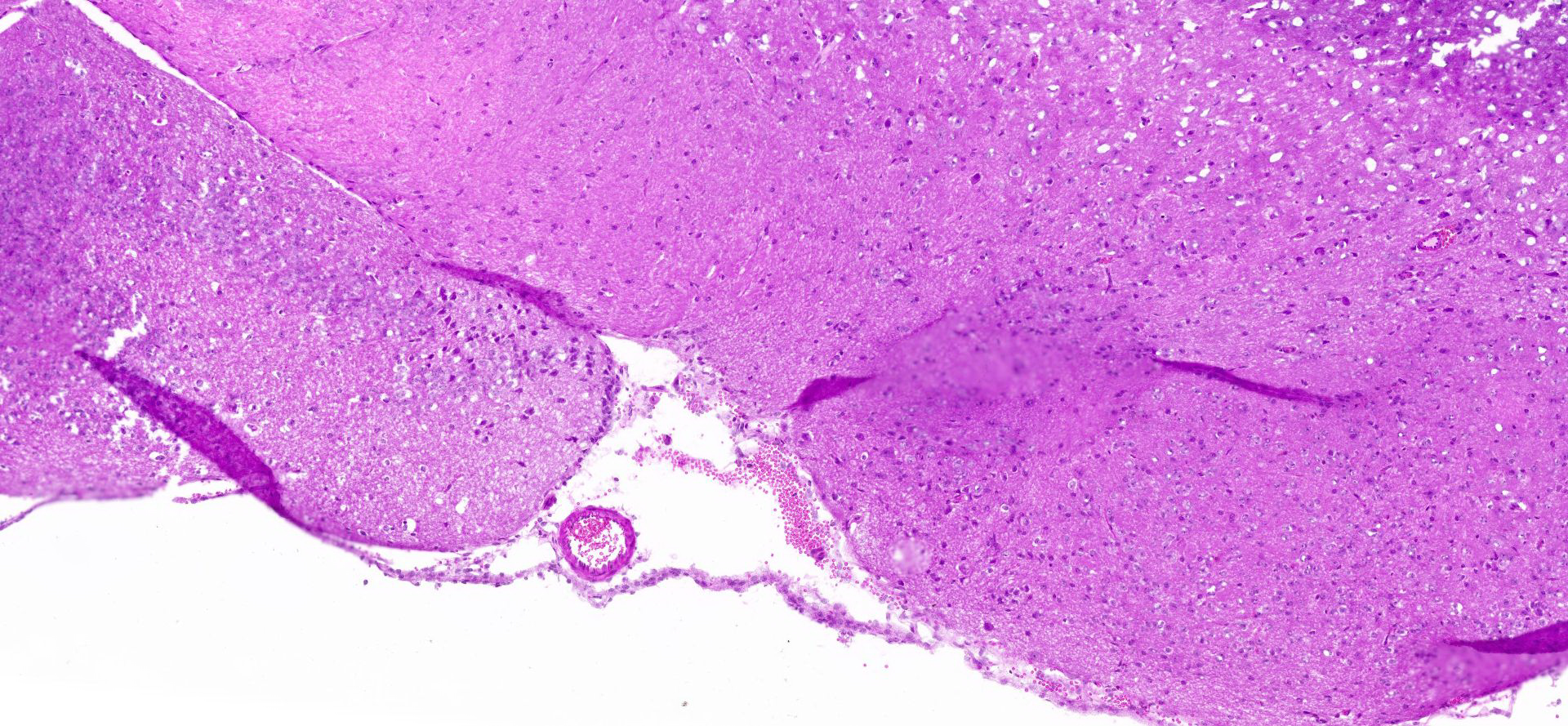

Supplement: S13 Fig — (TIF) [file ppat.1009256.s017.tif]

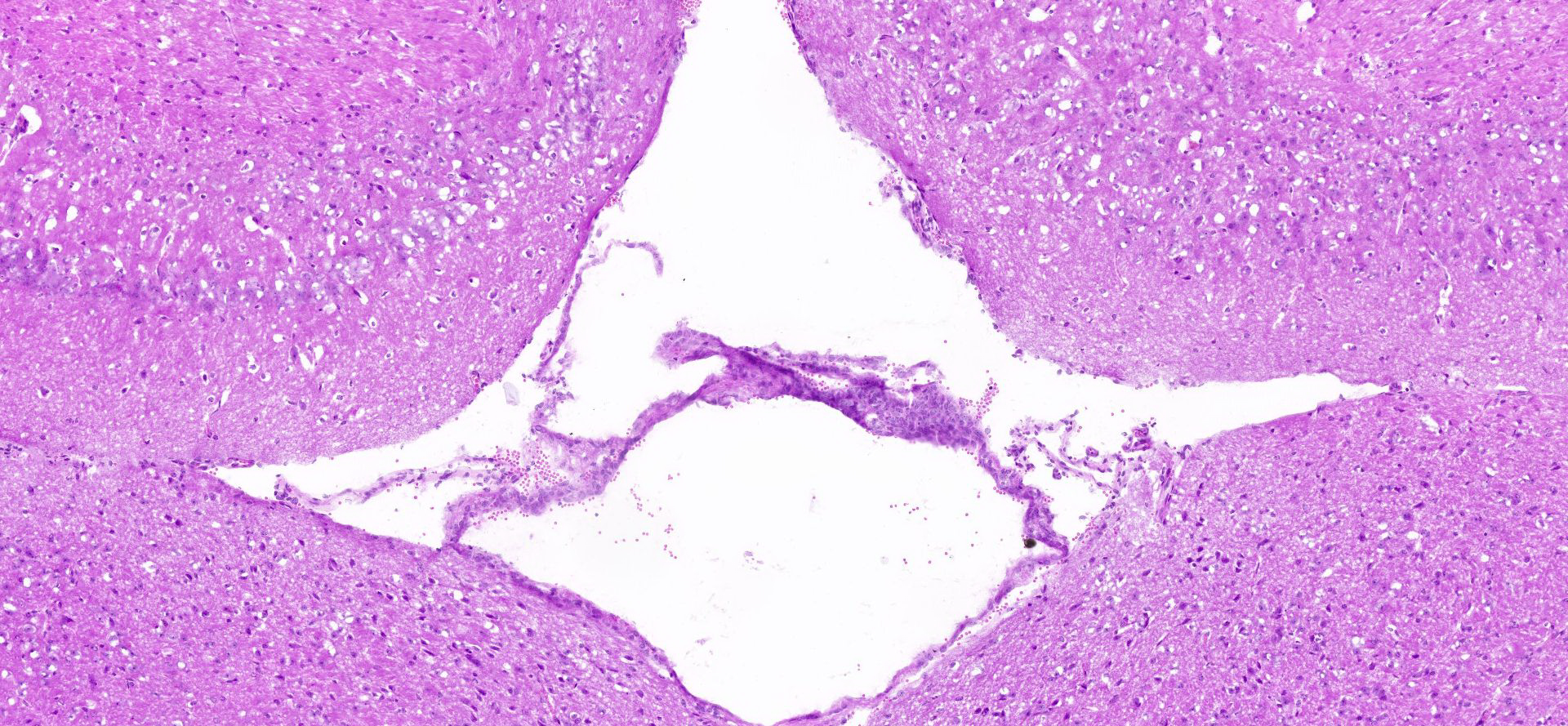

Supplement: S14 Fig — (TIF) [file ppat.1009256.s018.tif]

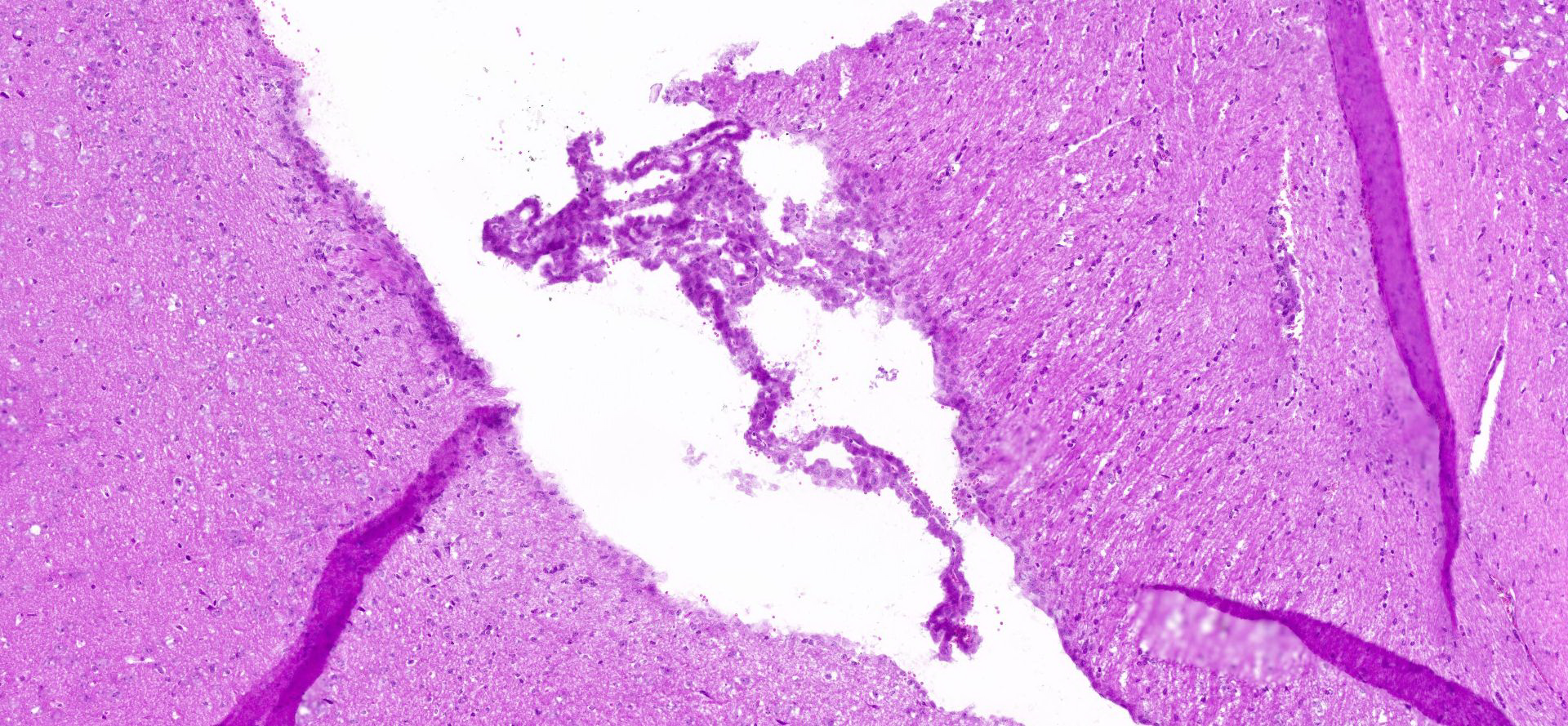

Supplement: S15 Fig — (TIF) [file ppat.1009256.s019.tif]

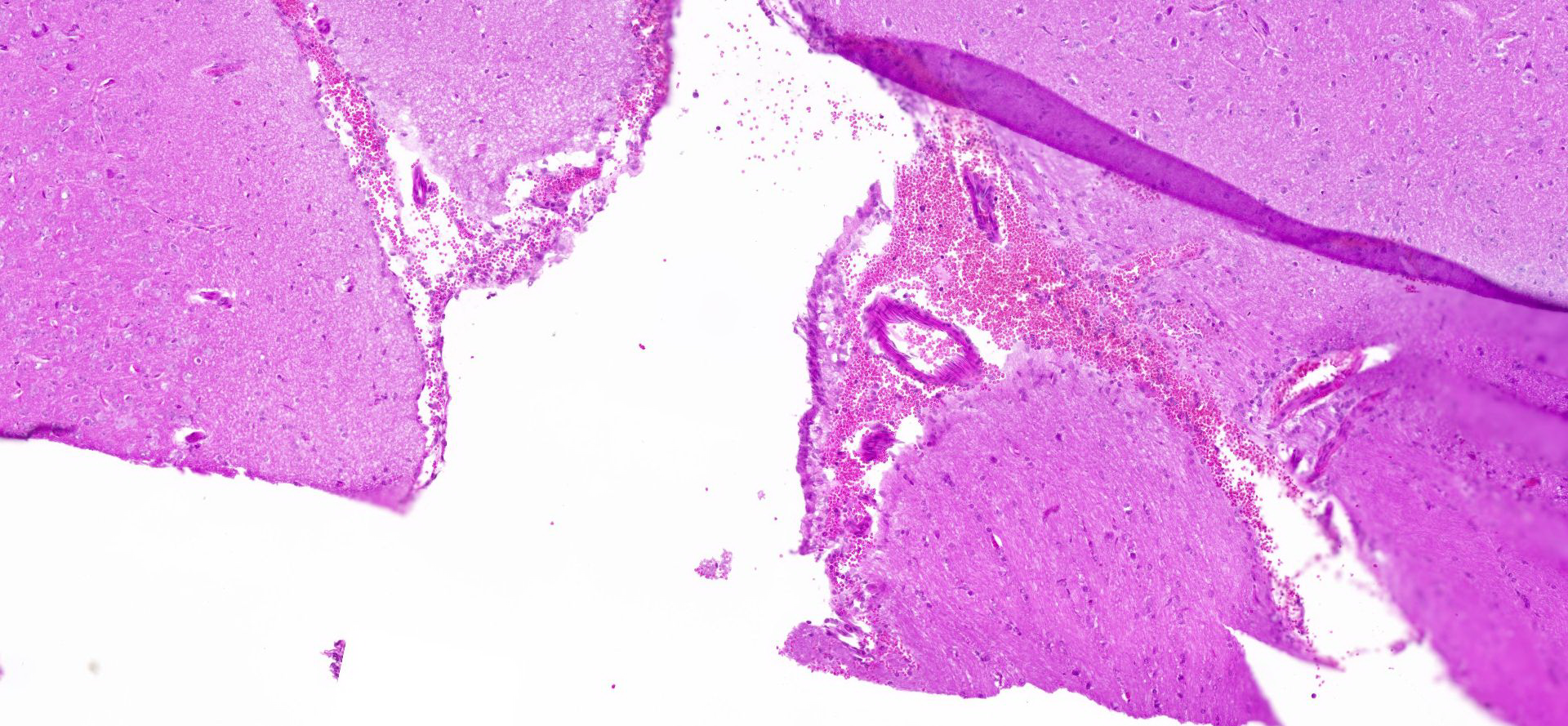

Supplement: S16 Fig — (TIF) [file ppat.1009256.s020.tif]

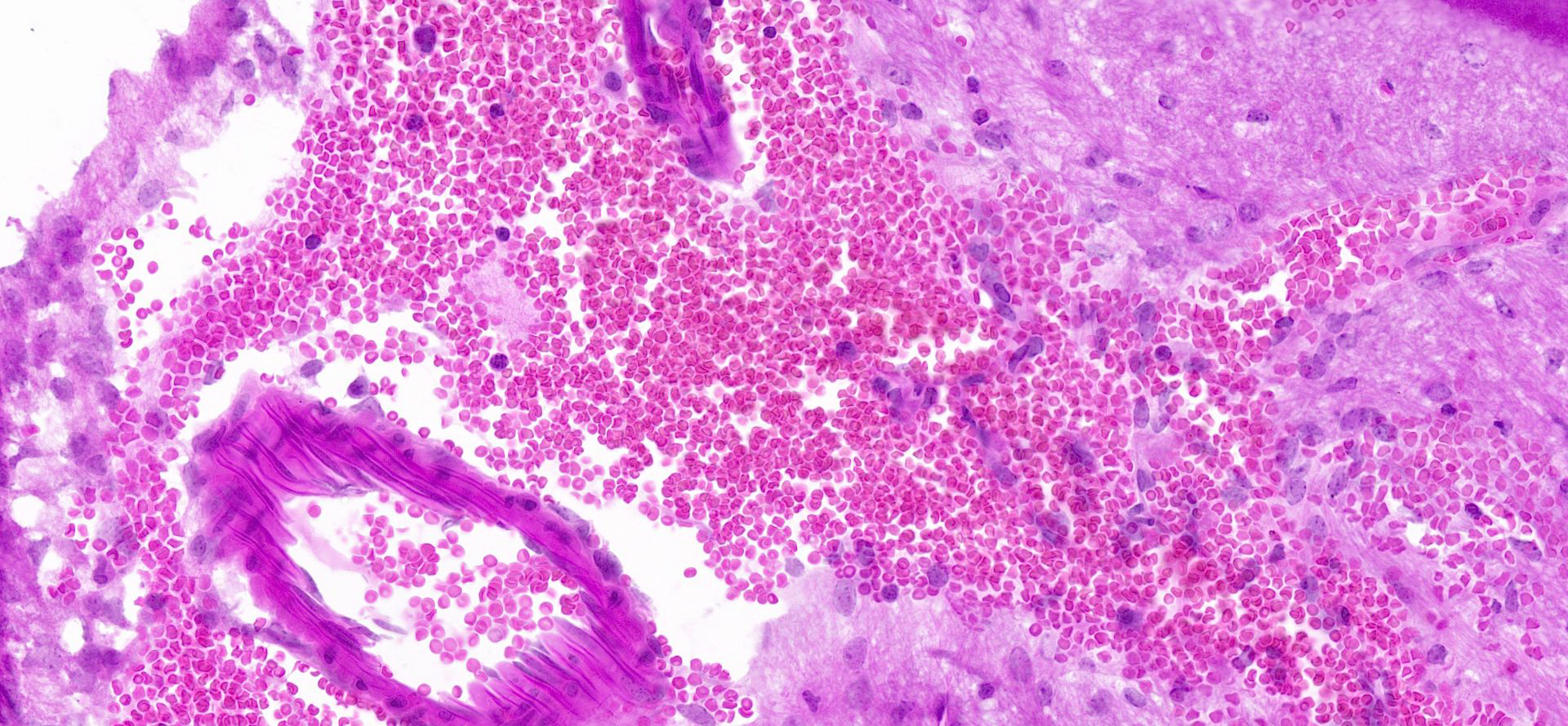

Supplement: S17 Fig — (TIF) [file ppat.1009256.s021.tif]

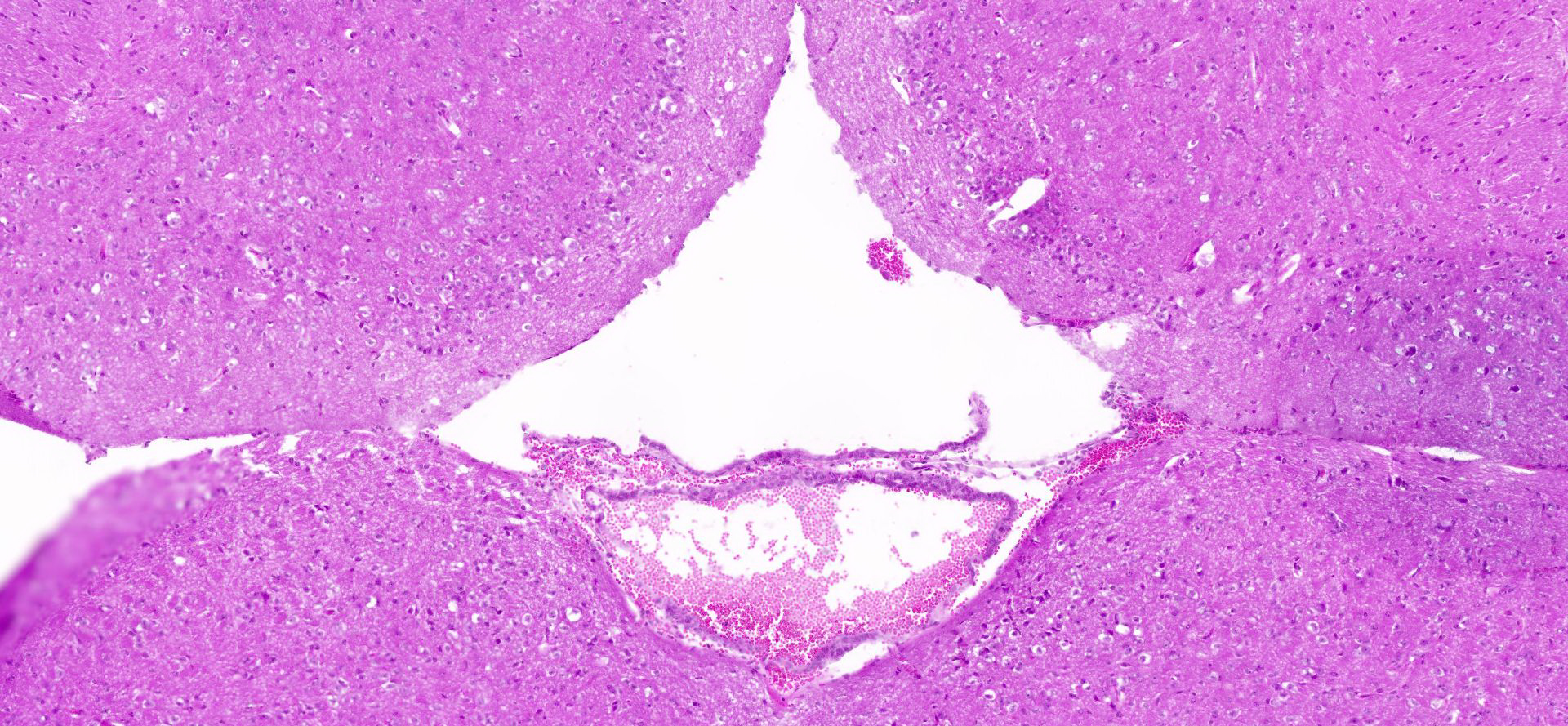

Supplement: S18 Fig — (TIF) [file ppat.1009256.s022.tif]

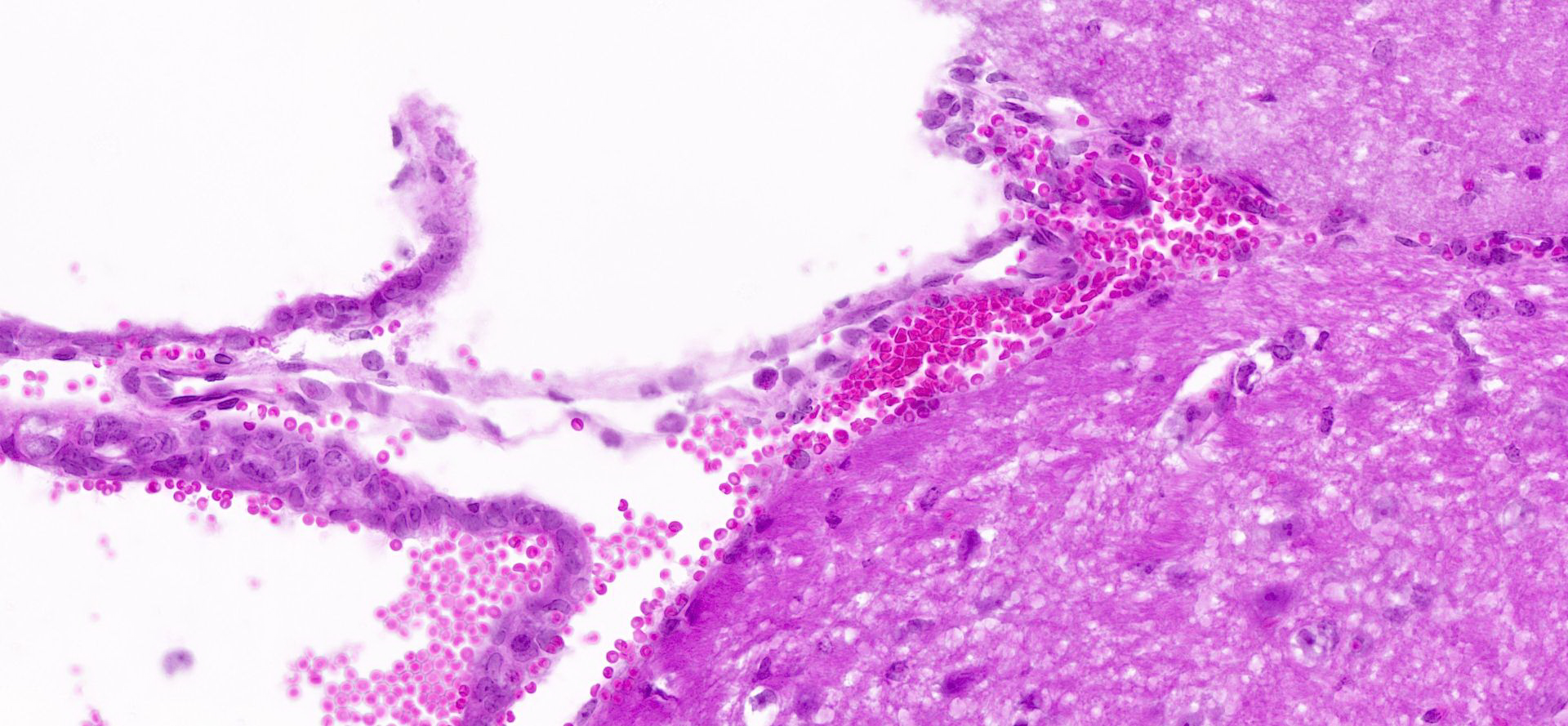

Supplement: S19 Fig — (TIF) [file ppat.1009256.s023.tif]

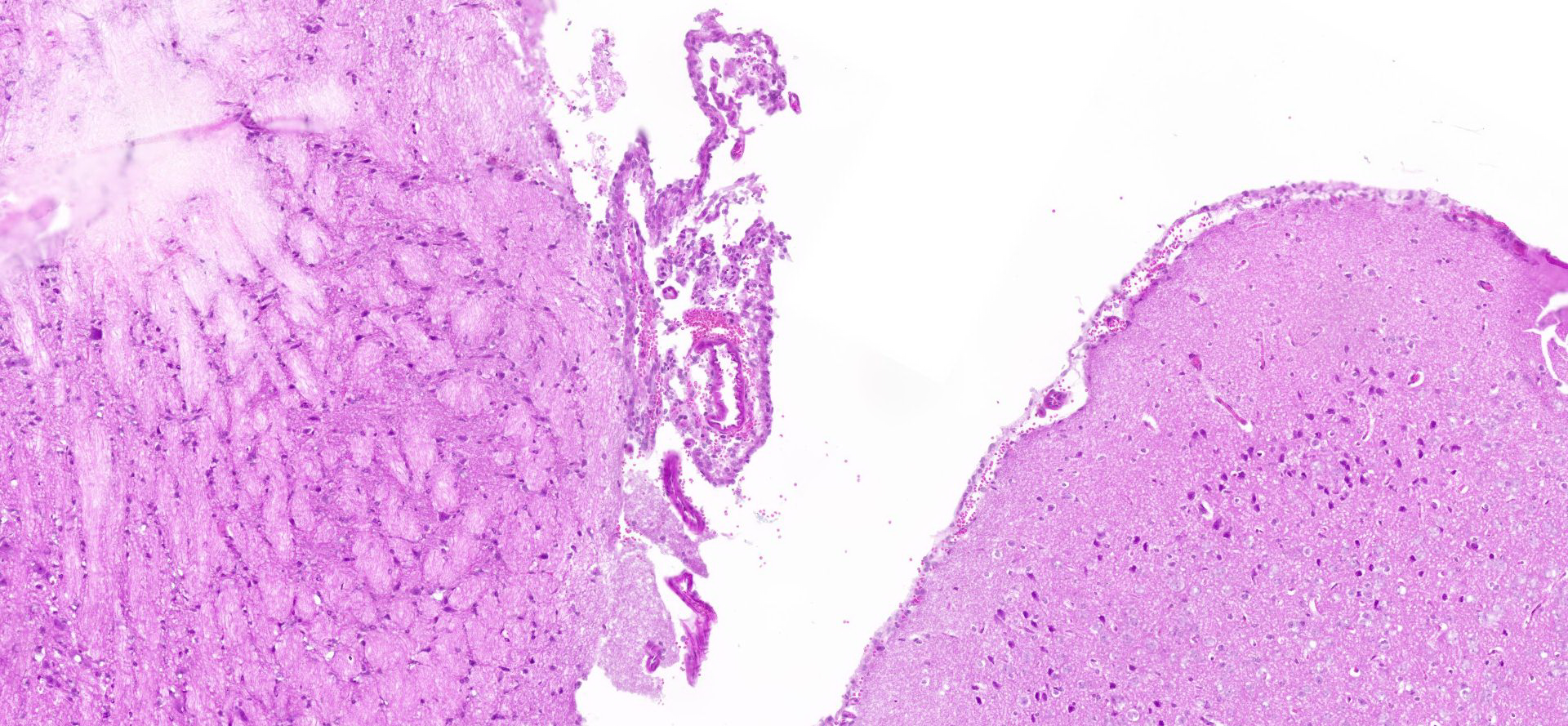

Supplement: S20 Fig — (TIF) [file ppat.1009256.s024.tif]

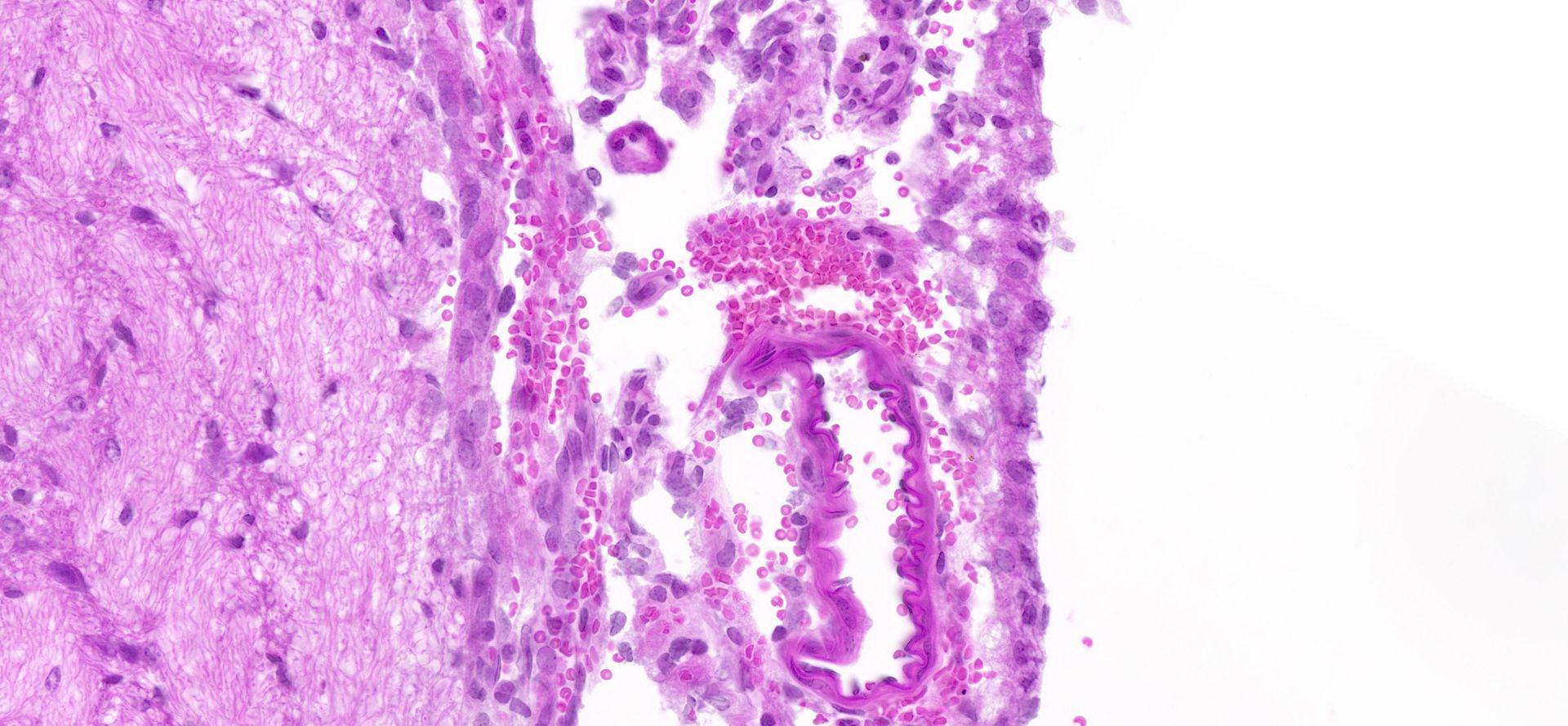

Supplement: S21 Fig — (TIF) [file ppat.1009256.s025.tif]
